# A new species of the paper wasp genus *Polistes* (Hymenoptera, Vespidae, Polistinae) in Europe revealed by morphometrics and molecular analyses

**DOI:** 10.3897/zookeys.400.6611

**Published:** 2014-04-11

**Authors:** Rainer Neumeyer, Hannes Baur, Gaston-Denis Guex, Christophe Praz

**Affiliations:** 1 Probsteistrasse 89, CH-8051 Zürich, Switzerland; 2 Abteilung Wirbellose Tiere, Naturhistorisches Museum der Burgergemeinde Bern, Bernastrasse 15, CH-3005 Bern, Switzerland; 3 Institute of Evolutionary Biology and Environmental Studies, Field Station Dätwil, University of Zurich, Winterthurerstrasse 190, CH-8057 Zürich, Switzerland; 4 Evolutionary Entomology, Institute of Biology, University of Neuchatel, Emile-Argand 11, CH-2000 Neuchâtel, Switzerland

**Keywords:** Cytochrome oxidase 1, ITS, DNA barcoding, principal component analysis, shape PCA, linear discriminant analysis, LDA ratio extractor, ratio spectrum, allometry, cryptic species, Switzerland

## Abstract

We combine multivariate ratio analysis (MRA) of body measurements and analyses of mitochondrial and nuclear data to examine the status of several species of European paper wasps (*Polistes* Latreille, 1802) closely related to *P. gallicus*. Our analyses unambiguously reveal the presence of a cryptic species in Europe, as two distinct species can be recognized in what has hitherto been considered *Polistes bischoffi* Weyrauch, 1937. One species is almost as light coloured as *P. gallicus*, and is mainly recorded from Southern Europe and Western Asia. The other species is darker and has a more northern distribution in Central Europe. Both species occur syntopically in Switzerland. Given that the lost lectotype of *P. bischoffi* originated from Sardinia, we selected a female of the southern species as a neotype. The northern species is described as *P. helveticus* **sp. n.** here. We also provide a redescription of *P. bischoffi* **rev. stat.** and an identification key including three more closely related species, *P. biglumis*, *P. gallicus* and *P. hellenicus*.

## Introduction

The paper wasp genus *Polistes* Latreille, 1802 (Hymenoptera, Vespidae, Polistinae) is an important model group for behavioral and evolutionary studies. It includes a large number of eusocial species that exhibit varied forms of social organization (West-Eberhard 1969). Moreover, its comparatively small colony size and exposed nests facilitate both field observations and experiments (e.g., Cervo et al. 2008). More than 220 species are currently recognized worldwide (Arens 2011, Buck et al. 2012, Nugroho et al. 2012: 72), ten of which occur in Europe (Arens 2011: 462, Carpenter 1997: 142, Castro and Dvořák 2009: 300). Three of them, namely *Polistes atrimandibularis* Zimmermann, 1930, *Polistes semenowi* Morawitz, 1889, and *Polistes sulcifer* Zimmermann, 1930, are social parasites (Cervo 2006, and references therein) and were considered as members of a distinct genus (or subgenus) *Sulcopolistes* Blüthgen, 1938 (Blüthgen 1961, Guiglia 1972), until Carpenter (1990) synonymized *Sulcopolistes* with *Polistes*. Later, phylogenetic analyses of one mitochondrial gene fragment showed that the three socially parasitic species formed a monophyletic group nested within other European *Polistes* (Choudhary et al. 1994: 33); the three social parasites constituted a monophyletic clade sister to a clade consisting of *Polistes dominula* (Christ, 1791) and *Polistes nimpha* (Christ, 1791).

Blüthgen (1943) proposed the subgeneric name *Leptopolistes* for several non-parasitic European species, including *Polistes associus* (Kohl, 1898), the type species of *Leptopolistes*, as well as *Polistes bischoffi* Weyrauch, 1937 and *Polistes gallicus* (Linnaeus, 1767). Males of these taxa share non-convex, immediately narrowing genae, as seen in dorsal view (Blüthgen 1943: 99; Guiglia 1972: 49), giving the male head a characteristically slender aspect. Currently, all European *Polistes* species are assigned to the subgenus *Polistes* (Carpenter 1996b), although the species formerly included in *Leptopolistes* species are still considered to be closely related (Carpenter 1997).

In fen rotational fallows (Gigon et al. 2010) at the shore of Lake Greifen [Greifensee] in the Swiss midlands Neumeyer et al. (2011) found a population of paper wasps that could not be assigned to any described species. This taxon is colored almost as light as *Polistes gallicus* (Linnaeus, 1767) and was therefore tentatively called “*Polistes* cf. *gallicus*” by Neumeyer et al. (2011). *Polistes gallicus* is quite common in Southern Europe, but it does not usually occur in wetlands and is not known as far north in Switzerland. The unidentified taxon from the Swiss midlands, however, shares an important trait (a reduced epicnemial carina) with another taxon that has hitherto been referred to as *Polistes bischoffi* Weyrauch, 1937 (e.g. Blüthgen 1961, Guiglia 1972, Mauss and Treiber 2004, Dvořák and Roberts 2006, Witt 2009), a common wetland-dweller in Switzerland and other countries of Central Europe.

To resolve the identity of the unidentified taxon from the Swiss wetlands, we examine its affinity to other European species using a combination of morphological, morphometric and molecular analyses. Recently, Buck et al. (2012) unraveled cryptic diversity in the Nearctic subgenus *Fuscopolistes* Richards, 1973 using multivariate morphometrics and DNA barcoding. In contrast to their study, we used a nuclear marker in addition to the mitochondrial marker and multivariate ratio analysis (MRA) instead of classic multivariate methods. MRA is a recently developed extension of principal component analysis (PCA) and linear discriminant analysis (LDA) that was specifically designed for the exploration of body measurements in a taxonomic context (Baur and Leuenberger 2011, László et al. 2013).

Our analyses lead to the recognition of two distinct species within what has been hitherto referred to as *Polistes bischoffi*; we review the information on the type material of *Polistes bischoffi*, and designate a neotype to settle the status of this species. *Polistes bischoffi* turns out to be the valid name of the unidentified taxon (“cf. *gallicus*”) found close to Zurich by Neumeyer et al. (2011); a new name is required for the species referred to as *Polistes bischoffi* by some authors (Blüthgen 1961, Guiglia 1972, Mauss and Treiber 2004, Dvořák and Roberts 2006, Witt 2009): *Polistes helveticus*, which is described here. Lastly, we provide an identification key that, in combination with available keys (Mauss and Treiber 2004, Dvořák and Roberts 2006, Witt 2009), will facilitate the identification of the Central European species.

## Material and methods

For the molecular and morphometric analyses we focus on the status of the two closely related morphs hitherto comprised under *Polistes bischoffi* (see introduction), as well as on the morphologically similar *Polistes gallicus*, and on their separation from other European *Polistes*. At this stage of the analyses, we deliberately avoid the concept of species and rather interprete them in the sense of operational taxonomic units, hereafter called “OTUs”. The OTUs are labeled with their valid taxonomic names (Carpenter 1996b), except for the two taxa hitherto comprised under *Polistes bischoffi* which are labeled in a manner that already anticipates the outcome of our study and our neotype designation. Detailed information on the taxonomic status of these names will be provided after the presentation of the results from the molecular and morphometric analyses.

### Molecular analyses

#### a) Species included

Ninety-nine specimens were included in the molecular analysis, representing eleven OTUs. In addition, two specimens each of *Vespula germanica* (Fabricius, 1793) and *Vespula vulgaris* (Linnaeus, 1758) were used to root the trees; sequences for *Polistes (Polistella) snelleni* Saussure, 1862 and *Polistes (Aphanilopterus) exclamans* Viereck, 1906 were downloaded from Genbank and used with the two species of *Vespula* to root the trees in analyses of the mitochondrial sequences. Most specimens were collected in 80% ethanol in the field, but we also included some specimens that were killed with ethyl acetate. For specimens collected before 2012, DNA was extracted from the mesosoma, leaving the legs, wings, head and metasoma as vouchers; for specimens collected in 2012 and 2013, as well as specimens selected as type specimens, DNA was extracted from one single leg to preserve a nearly intact specimen. Most specimens were collected in Switzerland, but we also included specimens form Croatia, France, Greece, Italy and Portugal (Table 1). All DNA extractions are deposited in the DNA bank of the Swiss Barcode of Life initiative (Swissbol; www.swissbol.ch).

**Table 1.** Locality information, voucher numbers and GenBank accession numbers for sequences used in this study.

Unit name	Voucher No	COX1	ITS1	Locality
Outgroup				
*Vespula germanica*	39	KJ415826	KJ415926	I, Crevoladossola
*Vespula germanica*	90	KJ415827	KJ415927	CH, Zürich
*Vespula vulgaris*	79	KJ415828	KJ415928	CH, Pfäffikon
*Vespula vulgaris*	89	KJ415829	KJ415929	CH, Zürich
*Polistes snelleni*	-	EF136457	-	-
*Polistes exclamans*	-	JN988655	-	USA, Florida, Archbold
Ingroup				
*Polistes associus*	7	KJ415830	KJ415930	HR, Rovinj
*Polistes associus*	286	KJ415831	KJ415931	CH, Losone
*Polistes biglumis*	21	KJ415832	KJ415932	CH, Val Müstair, Tschierv
*Polistes biglumis*	27	KJ415833	KJ415933	CH, Val Müstair, Sta. Maria
*Polistes biglumis*	28	KJ415834	KJ415934	CH, Val Müstair, Müstair
*Polistes biglumis*	29	KJ415835	KJ415935	CH, Val Müstair, Tschierv
*Polistes biglumis*	87	KJ415836	KJ415936	CH, Val Müstair, Tschierv
*Polistes bischoffi*	1	KJ415837	KJ415937	CH, Pfäffikon
*Polistes bischoffi*	22	KJ415838	KJ415938	CH, Pfäffikon
*Polistes bischoffi*	75	KJ415839	KJ415939	CH, Wetzikon
*Polistes bischoffi*	76	KJ415840	KJ415940	CH, Wetzikon
*Polistes bischoffi*	77	KJ415841	-	CH, Wetzikon
*Polistes bischoffi*	82	KJ415842	KJ415941	CH, Wetzikon
*Polistes bischoffi*	83	-	KJ415942	CH, Wetzikon
*Polistes bischoffi*	105	KJ415843	KJ415943	CH, Wetzikon
*Polistes bischoffi*	135	KJ415844	KJ415944	CH, Greifensee
*Polistes bischoffi*	136	KJ415845	KJ415945	CH, Greifensee
*Polistes bischoffi*	137	KJ415846	KJ415946	CH, Mönchaltorf
*Polistes bischoffi*	366	KJ415847	KJ415947	F, Corsica, Galeria
*Polistes dominula*	2	KJ415848	KJ415948	CH, Weiach
*Polistes dominula*	4	KJ415849	KJ415949	CH, Weiach
*Polistes dominula*	5	KJ415850	KJ415950	CH, Weiach
*Polistes dominula*	6	KJ415851	KJ415951	CH, Stallikon
*Polistes dominula*	15	KJ415852	KJ415952	CH, Wetzikon
*Polistes dominula*	16	KJ415853	KJ415953	CH, Wetzikon
*Polistes dominula*	25	KJ415854	KJ415954	CH, Val Müstair, Sta. Maria
*Polistes dominula*	26	KJ415855	KJ415955	CH, Val Müstair, Sta. Maria
*Polistes dominula*	30	KJ415856	KJ415956	CH, Grono
*Polistes dominula*	31	KJ415857	KJ415957	CH, Grono
*Polistes dominula*	32	KJ415858	KJ415958	CH, Grono
*Polistes dominula*	40	KJ415859	KJ415959	I, Masera
*Polistes dominula*	43	KJ415860	KJ415960	CH, Leuk
*Polistes dominula*	44	KJ415861	KJ415961	CH, Leuk
*Polistes dominula*	52	KJ415862	KJ415962	CH, Noville
*Polistes dominula*	54	KJ415863	KJ415963	CH, Meinier
*Polistes dominula*	58	KJ415864	-	CH, Cudrefin
*Polistes dominula*	63	KJ415865	KJ415965	CH, Cudrefin
*Polistes gallicus*	9	KJ415866	KJ415966	HR, Rovinj
*Polistes gallicus*	41	KJ415867	KJ415967	CH, Leuk
*Polistes gallicus*	42	KJ415868	KJ415968	CH, Leuk
*Polistes gallicus*	103	KJ415869	KJ415969	CH, Sant‘ Antonino
*Polistes gallicus*	108	KJ415870	KJ415970	P, Vila do Bispo
*Polistes gallicus*	115	KJ415871	KJ415971	CH, Villars-sous-Yens
*Polistes gallicus*	118	KJ415872	KJ415972	CH, San Vittore
*Polistes gallicus*	343	KJ415873	KJ415973	I, Cabras
*Polistes gallicus*	344	KJ415874	KJ415974	I, Macomer
*Polistes gallicus*	345	KJ415875	KJ415975	I, Scano di Montiferro
*Polistes gallicus*	346	KJ415876	KJ415976	I, Tadasuni
*Polistes gallicus*	347	KJ415877	KJ415977	I, Cabras
*Polistes gallicus*	348	KJ415878	KJ415978	I, Scano di Montiferro
*Polistes* sp. aff. *gallicus*	126	KJ415879*	-	GR, Ano Kotili
*Polistes* sp. aff. *gallicus*	129	KJ415880*	-	GR, Olympia
*Polistes hellenicus*	8	KJ415881	KJ415979	HR, Rovinj
*Polistes hellenicus*	10	KJ415882	KJ415980	HR, Rovinj
*Polistes hellenicus*	11	KJ415883	KJ415981	HR, Rovinj
*Polistes hellenicus*	88	-	KJ415982	HR, Rovinj
*Polistes hellenicus*	96	KJ415884	KJ415983	GR, Zacharo
*Polistes hellenicus*	101	KJ415885	KJ415984	HR, Vela Učka
*Polistes hellenicus*	102	KJ415886	KJ415985	HR, Vela Učka
*Polistes hellenicus*	133	KJ415887	-	GR, Avia bei Kalamata
*Polistes helveticus*	3	KJ415888	KJ415986	CH, Pfäffikon
*Polistes helveticus*	12	KJ415889	KJ415987	CH, Wetzikon
*Polistes helveticus*	13	KJ415890	KJ415988	CH, Wetzikon
*Polistes helveticus*	14	KJ415891	KJ415989	CH, Wetzikon
*Polistes helveticus*	17	KJ415892	KJ415990	CH, Wetzikon
*Polistes helveticus*	18	KJ415893	KJ415991	CH, Pfäffikon
*Polistes helveticus*	19	KJ415894	KJ415992	CH, Pfäffikon
*Polistes helveticus*	20	KJ415895	KJ415993	CH, Pfäffikon
*Polistes helveticus*	33	-	KJ415994	CH, Seegräben
*Polistes helveticus*	34	KJ415896	KJ415995	CH, Seegräben
*Polistes helveticus*	35	KJ415897	KJ415996	CH, Wetzikon
*Polistes helveticus*	46	KJ415898	KJ415997	CH, Noville
*Polistes helveticus*	47	KJ415899	KJ415998	CH, Noville
*Polistes helveticus*	48	KJ415900	KJ415999	CH, Noville
*Polistes helveticus*	49	KJ415901	KJ416000	CH, Noville
*Polistes helveticus*	50	KJ415902	KJ416001	CH, Noville
*Polistes helveticus*	51	KJ415903	KJ416002	CH, Noville
*Polistes helveticus*	78	KJ415904	KJ416003	CH, Pfäffikon
*Polistes helveticus*	81	KJ415905	-	CH, Wetzikon
*Polistes helveticus*	138	KJ415906	KJ416004	CH, Schwerzenbach
*Polistes helveticus*	139	KJ415907	-	CH, Schwerzenbach
*Polistes nimpha*	53	KJ415908	-	CH, Noville
*Polistes nimpha*	55	KJ415909	-	CH, Chabrey
*Polistes nimpha*	56	KJ415910	-	CH, Chabrey
*Polistes nimpha*	57	KJ415911	KJ416005	CH, Cudrefin
*Polistes nimpha*	59	KJ415912	-	CH, Cudrefin
*Polistes nimpha*	60	KJ415913	-	CH, Cudrefin
*Polistes nimpha*	61	KJ415914	-	CH, Cudrefin
*Polistes nimpha*	65	KJ415915	KJ416006	CH, Chabrey
*Polistes nimpha*	66	KJ415916	-	CH, Chabrey
*Polistes nimpha*	67	KJ415917	-	CH, Cudrefin
*Polistes nimpha*	68	KJ415918	-	CH, Cudrefin
*Polistes nimpha*	69	KJ415919	-	CH, Cudrefin
*Polistes nimpha*	85	KJ415920	-	CH, Meride
*Polistes nimpha*	86	KJ415921	-	I, Valsolda
*Polistes semenowi*	296	KJ415922	KJ416007	CH, Gampel
*Polistes sulcifer*	119	KJ415923	KJ416008	HR, Vela Učka
*Polistes sulcifer*	120	KJ415924	KJ416009	HR, Vela Učka
*Polistes sulcifer*	134	KJ415925	KJ416010	CH, Semione

* sequenced with UAE3/LepR instead of LepF/LepR

#### b) Lab protocols

Full lab protocols can be found in Praz et al. (2008). DNA was isolated using phenol-chloroform extractions; PCR reactions were performed with GoTaq polymerase (Promega) in a Biometra T1 thermocycler. PCR products were purified enzymatically using a mix of the enzymes exonuclease I (Fermentas) and FastAP thermosensitive alkaline phosphatase (Fermentas) and sequenced in both directions with the primers used in the original amplification using BigDye terminator technology (Applied Biosystems). Big Dye products were purified with Sephadex (GE Healthcare Life Sciences) and analyzed on a ABI-3500 DNA sequencer.

#### c) Markers and primers

We sequenced two fast-evolving genetic markers: the 600 bp fragment of the mitochondrial gene cytochrome oxidase 1 (COX1) used as an universal barcode (Hebert et al. 2003) and the nuclear marker ITS1; we chose ITS1 rather than ITS2 because preliminary analyses revealed that ITS2 was polymorphic in *Polistes bischoffi* and could not be sequenced directly.

For COX1 we used the universal primers LepF and LepR (Hebert et al. 2004) with the following conditions: an initial denaturation of 1 min at 94 °C, then six cycles of 1 min at 94 °C, 1.5 min at 45 °C, and 1.25 min at 72 °C, followed by 36 cycles of 1 min at 94 °C, 1.5 min at 51 °C, and 1.25 min at 72 °C, with a final step of 5 min at 72 °C. For specimens with degraded DNA, we used another universal forward primer, UAE3 (Zhang and Hewitt 1996) in combination with LepR to amplify a 400 bp fragment of the barcode. The conditions for this 400 bp fragment were as above, except that the extension time at 72 °C was 45 seconds in each cycle.

The presence of nuclear pseudogenes, or NUMTs, was carefully examined by visually detecting “ghost bands” on the agarose gel, and especially by detecting double peaks in the chromatograms. No indication of the presence of NUMTs was found in the specimens analyzed, with the exception of *Polistes nimpha*. For this OTU, double peaks were found in up to 20 nucleotide positions in every specimen, strongly suggesting the presence of NUMTs; no indels were found, and no stop codons were found in the translated amino acid sequence for these sequences, even when polymorphism was allowed, suggesting that the NUMTs were highly similar to the true mitochondrial sequences and thus of recent origin. The presence of NUMTs in *Polistes nimpha* was therefore unlikely to affect our results, especially given that *Polistes nimpha* was not the focus of our study, as it is not closely related to any of the main OTUs.

For ITS1, we used the primers CAS18sF1 and CAS5p8sB1d (Ji et al. 2003) to amplify a 700 bp fragment. For most specimens, the chromatograms were clean, without double peaks, indicating no within-specimen polymorphism in ITS1. In *Polistes dominula*, a few sites were polymorphic, and one insertion rendered the sequencing difficult in some specimens at position 550; in *Polistes nimpha*, several sites were polymorphic and insertions or deletions prevented direct sequencing in all specimens, except two (the numbers 57 and 65). Given that *Polistes nimpha* was not the focus of our study, we did not clone the PCR products to obtain clean sequences of the individual copies of ITS1, and merely included two specimens in our analysis.

#### d) Analyses

Genetic distances between each terminal were computed under the GTR model of nucleotide substitution in Paup 4.0b10 (Swofford 2002). We then performed maximum likelihood analyses of each marker separately using RAXML (Stamatakis et al. 2005), performing 1000 bootstrap replicates. For the mitochondrial marker, the first and second position were combined in one partition, while the third codon position constituted a second partition. For ITS1, we coded each insertion or deletion as an additional, binary character added as a separate partition, hereafter referred to as the “gap” partition; one insertion or deletion was considered as one character, regardless of the size of the indel. In total, the coding of the insertions and deletions resulted in 42 characters, 38 of which were parsimony informative and four of which were autapomorphic. We do not intend to unravel the phylogenetic relationships among the European species of *Polistes*, and therefore we do not present an analysis of a matrix combining both genes.

We applied a GTR + G model to each DNA partition; the gap partition was analyzed as a binary character with two states, with a gamma shape to accommodate rate heterogeneity. FigTree v1.3.1 (Rambaut 2009) was used to visualize the trees and produce the figures.

### Morphometrics

We restricted the morphometric analyses to the five most morphologically similar OTUs, namely *Polistes biglumis*, *Polistes bischoffi*, *Polistes gallicus*, *Polistes hellenicus*, and *Polistes helveticus*. For convenience, we refer hereafter to this group as the *Polistes gallicus*-group. We stress that we consider this group to be neither monophyletic nor taxonomically relevant.

#### a) Character selection and measurements

We measured a total of 266 specimens, most of them from Switzerland (158), but also some from Italy (30), Greece (24), Croatia (17), France (10), Germany (6), Slovakia (3), Turkey (3), Czech Republic (2), Liechtenstein (2), Austria (1), Azerbaijan (2), Uzbekistan or Tadjikistan (3), Mongolia (2), China (1), and Portugal (1). Sixteen characters were selected (Table 2) for measurements, most of them on the head and antenna, and two on the hind leg. Measurements were made on mounted specimens or parts (head, leg) of them using a pinholding device, permitting rotations around all three axes (X, Y, and Z). An Olympus SZH10 stereo-microscope equipped with eye-pieces Olympus GWH10X-D (with an eye-piece micrometer dividing 10 mm in 100 units) and Leica 10445111 (10x/21B) (with an eye-piece micrometer dividing 5 mm in 100 units) was used at several magnifications (Table 2). For terminology of morphological structures we followed Goulet and Huber (1993), occasionally also Richards (1973).

**Table 2.** Definition of distance measurements (* the Leica eye-piece micrometer dividing 5 mm in 100 units was used).

abbreviation	term	definition of measurement	magnification
cly.b	clypeus breadth	minimal distance between inner eye orbits	70×
eye.d	eye distance	minimal distance between inner eye orbits, dorsal view	50×
eye.h	eye height	height of eye in antero-lateral view	30×
flgfirst.l	first flagellomere length	length of first flagellomere, outer upper aspect	70×
flglast.b	terminal flagellomere breadth	breadth of terminal flagellomere, inner lateral aspect	*70×
flglast.l	terminal flagellomere length	length of terminal flagellomere (10th in female, 11th in male), inner lateral aspect	*70×
hea.b	head breadth	head breadth, dorsal view	30×
hea.h	head height	clypeal apex to anterior margin of median ocellus	30×
lof.l	lower face length	clypeal apex to lower margin of toruli	50×
msp.l	malar space	distance between lower eye orbit and mouth margin according to Arens (2011)	*70×
ool.l	lateral ocellus to eye distance	minimal distance between lateral ocellus and upper eye orbit	*70×
pol.l	lateral ocelli distance	minimal distance between lateral ocelli	*70×
scp.b	scape breadth	breadth of scape, dorsal view	*70×
scp.l	scape length	length of scape, inner lateral aspect	70×
tib3.b	metatibia breadth	breadth of metatibia, upper hind aspect	*70×
tib3.l	metatibia length	length of metatibia upper hind aspect	30×

#### b) Morphometric analysis

We applied the multivariate ratio analysis (MRA) of Baur and Leuenberger (2011) to our data. MRA comprises a set of tools for analyzing size and shape of body measurements in a multivariate mathematical framework that is entirely consistent with the customary usage of body lengths and ratios in taxonomic works (e.g., in descriptions, diagnoses). In systematic and taxonomic studies, MRA offers several advantages over conventional explorative multivariate methods, such as principal component analysis (PCA) and linear discriminant analysis (LDA). MRA removes biases from spurious contradictions in the results due to different definitions of size and shape. Furthermore, the numeric output of MRA can be used directly in the descriptive part of a taxonomic study. László et al. (2013) reviewed these issues in an application to parasitic wasps. Following Baur and Leuenberger (2011), we first calculated isometric size (isosize), defined as the geometric mean of all variables. We then performed a shape PCA (i.e., a principal component analysis in the space of all ratios) for evaluating how the morphometric pattern corresponds to the OTUs revealed in the molecular analyses. In order to decide how many components to retain we inspected the scree graph (Rencher 2002: 398–399). We also plotted isosize against shape PCs, because the correlation of size with shape is a measure of the amount of allometry in the data. Two graphical tools, the PCA ratio spectrum and allometry ratio spectrum respectively, were also employed in some cases. Finally, we used the LDA ratio extractor to extract the best ratios, and calculated the standard distance as well as the measure *δ*.

The R language and environment for statistical computing was used for data analysis (R Development Core Team 2013; version 3.0.1). For the above methods we employed slightly modified versions of the R-scripts provided by Baur and Leuenberger (2011, under “Supplementary material”). Scatterplots were generated with the package “ggplot2” (Wickham 2009).

### Taxonomic treatment, voucher and type specimens

For taxonomy and classification we followed Carpenter (1996b). Abbreviations used for specimen depositories and other institutions or private collections cited in this study are given in Table 3. Stack-photographs of mounted specimens were taken with a Keyence VHX-2000 digital microscope at the NMBE. All known *Polistes* collections in Switzerland (CH), as well as several collections elsewhere (Table 3), have been examined by one of the authors (RN). We also examined the relevant type material.

**Table 3.** Abbreviations of depositories (museums and private collections) and other institutions. "CH" means Switzerland.

abbreviation	full name
museums and other institutions	
**AMNH**	American Museum of Natural History, New York, USA
**BNM**	Bündner Naturmuseum, Chur, CH
**CSCF**	Swiss Biological Records Center, Neuchâtel, CH
**ETHZ**	Eidgenössische Technische Hochschule, Zürich, CH
**FMLT**	Fundación Miguel Lillo, Tucumán, Argentina
**HNHM**	Hungarian Natural History Museum, Budapest, Hungary
**HUMCZ**	Harvard University Museum of Comparative Zoology, Cambridge, MA, USA
**LSL**	Linnean Society of London, GB
**MACN**	Museo Argentino de Ciencias Naturales, Buenos Aires, Argentina
**MCHNS**	Musée cantonal d‘histoire naturelle, Sion, CH
**MCSNL**	Museo cantonale di storia naturale, Lugano, CH
**MCSNV**	Museo Civico di Storia Naturale, Verona, Italy
**MFNB**	Museum für Naturkunde, Berlin, Germany
**MHNF**	Musée d‘histoire naturelle, Fribourg, CH
**MHNG**	Muséum d‘histoire naturelle de la Ville de Genève, CH
**MHNL**	Museo de Historia Natural, Lima, Peru
**MHNN**	Muséum d‘histoire naturelle de Neuchâtel, CH
**MMML**	Mĕstské muzeum Mariánské Láznĕ, Czech Republic
**MNHN**	Muséum national d‘histoire naturelle, Paris, France
**MSNM**	Museo Civico di Storia Naturale, Milano, Italy
**MSNUF**	Museo di Storia Naturale dell‘Università, Firenze, Italy
**MSNV**	Museo di Storia Naturale, Venezia, Italy
**MZL**	Musée cantonal de zoologie, Lausanne, CH
**NBC**	Naturalis Biodiversity Center, Leiden, Netherlands
**NHMB**	Naturhistorisches Museum Basel, CH
**NHMW**	Naturhistorisches Museum Wien, Austria
**NMBE**	Naturhistorisches Museum der Burgergemeinde Bern, CH
**NML**	Natur-Museum Luzern, CH
**NMSO**	Naturmuseum Solothurn, CH
**NMTG**	Naturmuseum Thurgau, Frauenfeld, CH
**NMW**	Naturmuseum Winterthur, CH
**SDEI**	Senckenberg Deutsches Entomologisches Institut, Müncheberg, Germany
**ZIN**	Zoological Institute of the Russian Academy of Sciences, Saint Petersburg, Russia
**ZMUZ**	Zoologisches Museum der Universität Zürich, CH
**ZSM**	Zoologische Staatssammlung München, Germany
Private collections	
**AK**	Albert Krebs
**AR**	André Rey
**CM**	Christian Monnerat
**CSE**	Christian Schmid-Egger
**ES**	Erwin Steinmann
**FA**	Felix Amiet
**GA**	Georg Artmann-Graf
**HT**	Hansueli Tinner
**IK**	Igor Kramer
**IS**	Irene Salzmann
**JF**	Jakob Forster
**JS**	Jan Smit
**LD**	Libor Dvořák
**MH**	Mike Herrmann
**PS**	Peter Schär
**RN**	Rainer Neumeyer
**WA**	Werner Arens
**WS**	Wolfgang Schlaefle
**YC**	Yannick Chittaro

## Data resources

The morphometric data underpinning the analyses reported in this paper as well as a series of images showing the exact character definitions are deposited in the Dryad Digital Repository at http://doi.org/10.5061/dryad.9b8tt.

## Results

### Molecular analyses

#### a) Sequencing

Of the 99 ingroup specimens included, complete COX1 sequences were obtained for 96 specimens, and ITS sequences for 80 specimens (Table 1). This difference is due to 12 ITS sequences of *Polistes nimpha* that were polymorphic and excluded, as well as some specimens with degraded DNA, which could be sequenced for the shorter mitochondrial fragment but not for ITS1.

#### b) COX1

**Figure 1.**
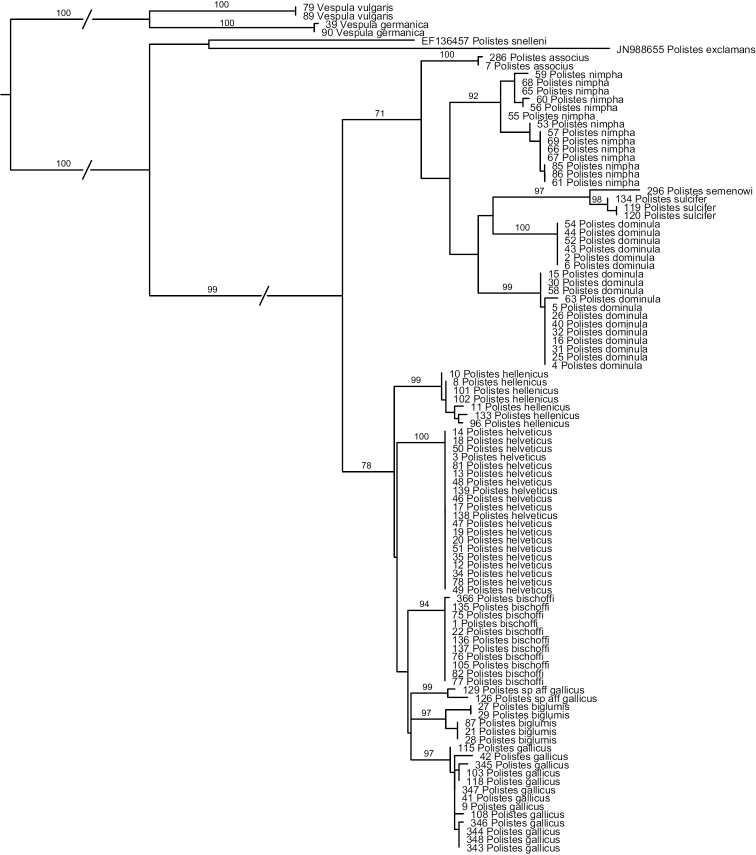
Phylogenetic tree based on maximum likelihood analysis of sequences of the mitochondrial gene cytochrome oxidase 1 (COX1); numbers shown at nodes are bootstrap values based on 1000 bootstrap replicates performed in RAxML v.7.0.4. Branches interrupted by an oblique line have been shortened for better graphic representation.

Analyses of the COX1-sequences (Fig. 1) reveal that *Polistes helveticus* and *Polistes bischoffi* represent two distinct, well-supported clades (Bootstrap support, hereafter BS, of 100 and 94%, respectively). Sequences of all included specimens of *Polistes bischoffi*, including the 10 specimens from Switzerland and one specimen from Corsica, were absolutely identical (genetic distance of 0); similarly, sequences of the 20 specimens of *Polistes helveticus* were identical. The genetic distance between these two clades was 2.6%. The relationship between these two clades, as well as the relationships among the species of the *gallicus*-group, were not resolved.

More generally, most OTUs included in this study were recovered as monophyletic with high bootstrap support >90%, with the exception of *Polistes dominula*. Sequences for this OTU formed two well-supported clades (see below). The two specimens identified as *Polistes gallicus* by Arens (2011) did not appear closely related to other specimens of *Polistes gallicus*. For this reason, this taxon is simply referred to as *Polistes* sp. aff. *gallicus*.

Maximal within-OTU genetic distances were 0.2% for *Polistes associus*, 0.3% for *Polistes sulcifer*, 1.0% for *Polistes hellenicus* and for *Polistes gallicus*, 1.06% for *Polistes* sp. aff. *gallicus*, and 1.4% for *Polistes biglumis*. Within OTU-distances were higher for *Polistes nimpha* (2.4%) and especially for *Polistes dominula* (up to 4.9%; see below). For *Polistes nimpha*, although two weakly supported clades are revealed within this OTU (Fig. 1), the ranges of distance within (0–0.6% and 0–0.7%) and between these clades (0.4–2.4%) overlapped. In contrast, sequences for *Polistes dominula* formed two distinct clades that did not overlap. All sequences within the first clade were identical, thus the distance within this clade was equal to 0. In the second clade, the distances ranged from 0 to 0.67%; the distances between these two clades were between 3.6 and 4.9%. These two clades were weakly associated with geographic location: specimens originating from western Switzerland (Geneva, Valais and one location in Vaud) and from one site close to Zurich formed one clade, whereas specimens originating from the Grisons, from one location in Vaud and from the southern parts of the canton of Zurich formed the other clade; specimens from one locality in Zurich were distributed in both clades.

The minimal distance between two OTUs was 2.6%, observed between *Polistes helveticus* and *Polistes bischoffi*, as indicated above, as well as between the two included social parasites, *Polistes semenowi* and *Polistes sulcifer*.

#### c) ITS1

**Figure 2.**
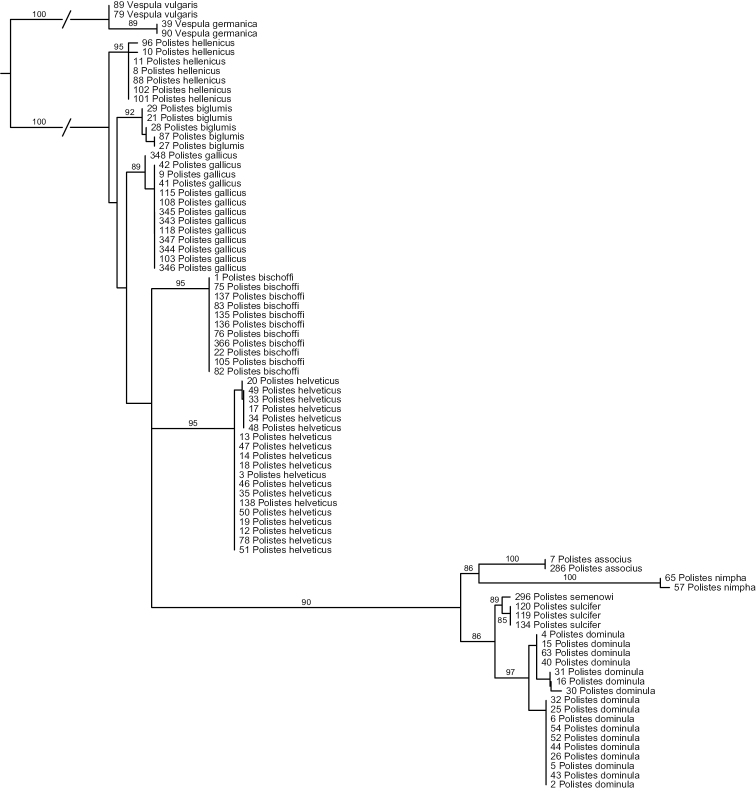
Phylogenetic tree based on maximum likelihood analysis of sequences of the nuclear marker ITS1; numbers shown at nodes are bootstrap values based on 1000 bootstrap replicates performed in RAxML v.7.0.4. Branches interrupted by an oblique line have been shortened for better graphic representation.

Analyses of ITS1 (Fig. 2) again strongly suggest that *Polistes helveticus* and *Polistes bischoffi* represent two distinct, well supported clades (both with BS of 95%). Sequences for all of the eleven specimens of *Polistes bischoffi*, including one specimen from Corsica, were identical; within *Polistes helveticus*, the genetic distance was 0.17% due to one single polymorphic site. The genetic distances between both clades were between 2.23% and 2.37%. The relationship between these two species, as well as the relationships among the different species of the *gallicus*-group, were not resolved.

All other OTUs were recovered as well supported clades, with bootstrap supports > 85% (Fig. 2). No sequence of ITS1 could be obtained for the two specimens of *Polistes* sp. aff. *gallicus* from Greece. The two clades observed in analyses of the mitochondrial marker in *Polistes dominula* were not recovered in analyses of ITS1, although maximal within-OTU distances were comparatively high for this OTU (0.77%). However, no distance correlation between ITS1 and COX1 was observed; for example, some specimens exhibiting high mitochondrial distances (eg, numbers 5 and 43) had identical ITS1 sequences. Other within-OTU genetic distances were as follows: 0% for *Polistes sulcifer*, *Polistes associus* and *Polistes gallicus*; 0.24% for *Polistes biglumis*; 0.32% for *Polistes hellenicus*.

The smallest interspecific distance in ITS1 sequences was 0.8%, between *Polistes biglumis* and *Polistes hellenicus*; the maximal distance in our ingroup was 11.5%, observed between *Polistes nimpha* and *Polistes biglumis*. The minimum distance between *Polistes bischoffi* and any other OTU was 1.72%, between *Polistes bischoffi* and *Polistes biglumis*.

### Multivariate ratio analysis (MRA) of the *gallicus*-group

**Figure 3.**
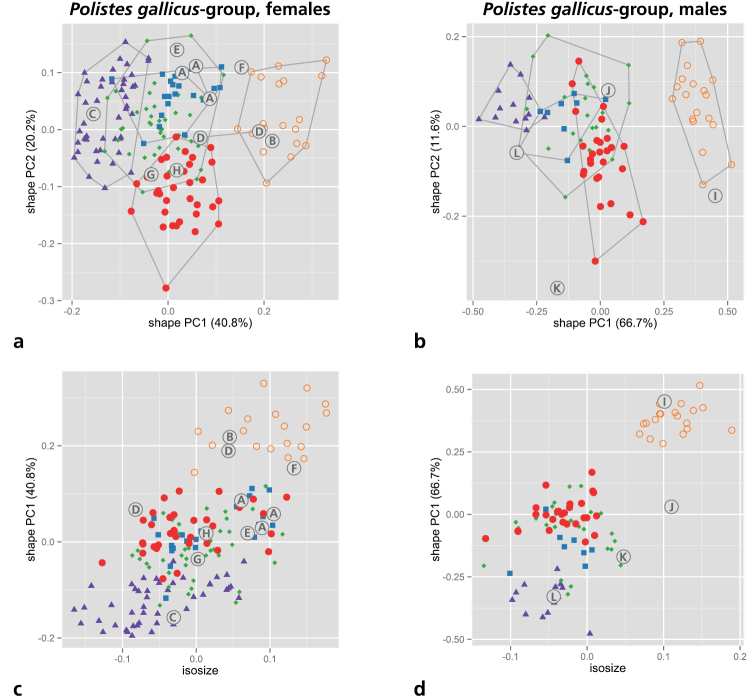
Shape PCA of all five OTUs of the *Polistes gallicus*-group. **a, b** Scatterplot of first against second shape PC **a** females **b** males **c, d** Scatterplot of isosize against first shape PC **c** females **d** males. Symbols: orange circles = *Polistes biglumis*, purple triangles = *Polistes bischoffi*, green diamonds = *Polistes gallicus*, blue rectangles = *Polistes hellenicus*, red dots = *Polistes helveticus*. In parentheses, the variance explained by each shape PC. Encircled upper case letters show the position of the type specimens of various nominal taxa: (**A**) *Polistes bimaculatus pamirensis* Zirngiebl, 1955, syntypes; (**B**) *Polistes bimaculatus nigrinotum* Zirngiebl, 1955, holotype; (**C**) *Polistes bischoffi* Weyrauch, 1937, neotype; (**D**) *Polistes foederatus obscuricornis* Mader, 1936, syntypes; (**E**) *Polistes omissus ordubadensis* Zirngiebl, 1955, holotype; (**F**) *Polistes omissus kaszabi* Giordani Soika, 1970, holotype; (**G**) *Polistes foederatus albellus* Giordani Soika, 1976, paratype; (**H**) *Polistes helveticus* Neumeyer sp. n., holotype; (**I**) *Polistes dubius* Kohl, 1898, lectotype; (**J**) *Polistes foederatus* Kohl, 1898, holotype; (**K**) *Polistes gallicus mongolicus* Buysson, 1911, syntype; (**L**) *Polistes hellenicus* Arens, 2011, holotype.

As mentioned above in material and methods, we restricted the MRA to the five OTUs of the *gallicus*-group (s. Table 4 for an overview of measurements). We first performed a shape PCA to see how well the monophyletic OTUs recovered by molecular analyses (Figs 1 and 2) are supported by morphometric variation. A PCA is convenient because it does not require *a priori* assignment of OTUs to particular groups but assumes instead that all OTUs belong to one single group. A PCA thus avoids circular reasoning with respect to particular groupings (see Peters and Baur 2011). According to the scree graph (not shown), only the first and second shape PC were relevant, comprising more than 60% of the total variation. Scatterplots of the two axes gave a very similar result for both sexes (Figs 3a, b). *Polistes biglumis* was clearly separable from the other species along the first shape PC. The other OTUs were much closer, with *Polistes bischoffi* and *Polistes helveticus* still being rather distinct. The ranges of the two remaining OTUs, *Polistes gallicus* and *Polistes hellenicus*, were entirely overlapping. A scatterplot of isosize and the first shape PC revealed a strong correlation between size and shape (Figs 3c, d). This was mainly caused by the presence of *Polistes biglumis*, which was clearly the largest OTU in both sexes. The others were largely overlapping in their size ranges.

**Table 4.** Summary of measurements (in µm) used for morphometric analysis. For abbreviations, see Table 1.

***Polistes biglumis***								
	females n=19				males n=20			
	Min	Max	Mean	SD	Min	Max	Mean	SD
cly.b	1286	1586	1453.4	84.23	1157	1371	1241.4	52.62
eye.d	1540	1880	1704.2	98.79	1480	1760	1602	65.82
eye.h	2033	2500	2268.4	131.22	2067	2433	2280	80.5
flgfirst.l	829	1000	932.3	48.06	1000	1129	1081.4	37.11
flglast.b	286	343	315	15.95	157	186	172.9	9.15
flglast.l	300	393	334.6	22.75	293	421	364.3	37.08
hea.b	2933	3500	3238.6	175.07	3067	3533	3261.7	98.1
hea.h	2600	3033	2805.3	136.2	2467	2833	2643.3	75.78
lof.l	1580	1920	1751.6	94.83	1540	1840	1695	59.78
msp.l	371	514	445.5	34.85	371	436	403.6	22.53
ool.l	514	629	578.6	31.59	471	571	529.3	29.03
pol.l	264	379	325.2	33.53	279	386	326.4	22.72
scp.b	379	486	444	28.3	450	564	512.1	26.74
scp.l	1157	1400	1281.2	71.75	1129	1314	1202.1	49.55
tib3.b	507	650	585	40.05	521	614	577.9	29.3
tib3.l	2900	3767	3387.7	250.73	3000	3733	3428.3	163.04
***Polistes bischoffi***								
	females n=46				males n=13			
	Min	Max	Mean	SD	Min	Max	Mean	SD
cly.b	1057	1386	1216.5	90.3	943	1100	983.5	44.74
eye.d	1300	1640	1453.9	80.76	1280	1480	1352.3	57.47
eye.h	1833	2333	2069.6	135.32	1900	2167	2056.4	87.54
flgfirst.l	671	886	781.1	53.03	871	1000	944	30.56
flglast.b	257	314	286.5	14.85	150	171	158.2	7.05
flglast.l	279	386	320.2	18.83	400	471	425.3	23.26
hea.b	2533	3167	2842	163.52	2667	3033	2864.1	100.43
hea.h	2167	2767	2447.8	152.45	2167	2433	2305.1	81.47
lof.l	1340	1720	1516.1	102.99	1460	1620	1520	48.99
msp.l	250	350	289.4	22.57	171	207	187.4	10.97
ool.l	429	529	472.8	26.92	379	479	417	28.35
pol.l	264	357	321.9	21.63	293	364	319.8	20.24
scp.b	314	414	365.1	27.32	379	450	416.5	20.07
scp.l	986	1271	1113.7	70.5	943	1100	1041.8	46.46
tib3.b	393	600	486	46.19	429	500	470.9	24.99
tib3.l	2600	3633	3072.5	256.69	2767	3233	3061.5	156.26
***Polistes gallicus***								
	females n=42				males n=25			
	Min	Max	Mean	SD	Min	Max	Mean	SD
cly.b	1143	1471	1290.5	74.11	900	1186	1026.3	59.29
eye.d	1420	1720	1560.5	73.58	1200	1560	1419.2	75.38
eye.h	1900	2533	2134.1	125.26	1833	2267	2064	118.21
flgfirst.l	714	929	795.6	46.85	886	1114	975.4	48.36
flglast.b	271	336	303.2	14.13	136	179	160.6	12.39
flglast.l	286	357	313.6	18.92	329	457	385.4	33.02
hea.b	2733	3400	3017.5	149.3	2633	3233	2921.3	149.96
hea.h	2333	2900	2556.3	134.24	2167	2733	2401.3	122.66
lof.l	1420	1820	1583.8	89.14	1420	1820	1575.2	87.99
msp.l	293	393	333.3	24.93	186	307	243.1	27.85
ool.l	457	571	512.4	32.02	357	521	450.3	28.31
pol.l	279	371	333.3	21.32	271	371	308.9	27.53
scp.b	343	436	383.7	22.1	379	471	428	22.77
scp.l	1057	1343	1172.4	61.21	914	1171	1054.3	62.13
tib3.b	471	607	525.7	37.5	436	600	493.1	37.6
tib3.l	2767	3733	3211.9	236.94	2600	4000	3212	269.92
***Polistes hellenicus***								
	females n=21				males n=9			
	Min	Max	Mean	SD	Min	Max	Mean	SD
cly.b	1171	1457	1313.6	87.19	957	1057	1014.3	34.99
eye.d	1440	1720	1568.6	92.43	1280	1540	1422.2	98.71
eye.h	2000	2400	2188.9	143.89	1933	2200	2055.6	92.8
flgfirst.l	743	929	831.3	51.63	871	986	941.3	40.05
flglast.b	279	336	299	15.2	143	171	155.6	8.58
flglast.l	271	371	314.3	27.94	364	414	391.3	18.48
hea.b	2767	3300	3036.5	172.85	2600	2967	2837	112.35
hea.h	2367	2867	2603.2	158.43	2200	2467	2377.8	83.33
lof.l	1460	1820	1613.3	113.37	1460	1640	1566.7	58.31
msp.l	279	400	341.5	34	179	257	219	22.87
ool.l	457	564	517.7	32.62	414	514	462.7	30.88
pol.l	271	357	322.8	20.78	286	336	302.4	20.52
scp.b	364	436	396.9	18.98	379	443	420.6	22.99
scp.l	1086	1286	1181	72.19	957	1100	1042.9	40.41
tib3.b	500	614	544.6	32.25	464	521	496	20.55
tib3.l	3033	3767	3360.3	246.45	3033	3333	3225.9	124.47
***Polistes helveticus***								
	females n=34				males n=27			
	Min	Max	Mean	SD	Min	Max	Mean	SD
cly.b	1071	1457	1254.2	83.36	914	1057	994.2	37.64
eye.d	1420	1780	1538.8	79.99	1280	1600	1425.2	71.6
eye.h	1800	2333	2045.1	129.2	1800	2133	1998.8	82.42
flgfirst.l	657	871	780.7	49.47	857	986	928	33.03
flglast.b	264	321	295	16.82	129	193	161.6	12.07
flglast.l	300	407	327.9	23.81	279	457	346.8	37.76
hea.b	2567	3233	2866.7	155.27	2633	3033	2893.8	90.13
hea.h	2267	2833	2521.6	147	2133	2467	2355.6	71.61
lof.l	1380	1780	1552.4	100.79	1400	1620	1547.4	54.95
msp.l	293	429	357.4	29.92	214	293	249.5	20.2
ool.l	450	607	515.1	28.07	386	536	450	31.13
pol.l	293	414	348.3	27.75	279	379	328.6	24.82
scp.b	343	457	387.6	31.52	386	464	430.2	16.38
scp.l	1000	1300	1129.8	70.63	943	1086	1028	33.26
tib3.b	421	600	506.1	48.06	429	536	487.3	27.83
tib3.l	2667	3567	3049	223.27	2800	3300	3080.2	142.1

**Figure 4.**
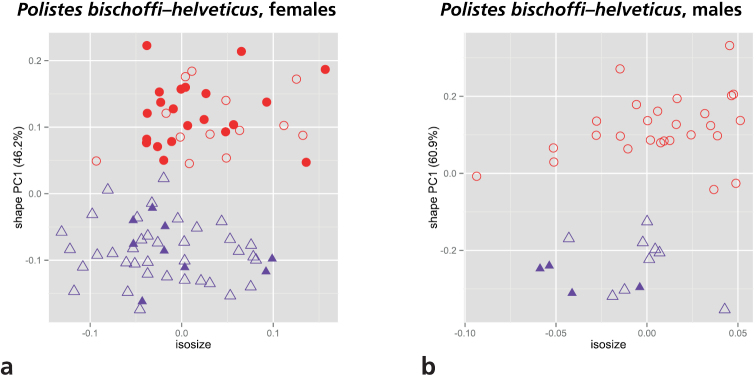
Scatterplot of first shape PC against isosize for comparison of *Polistes bischoffi* with *Polistes helveticus*: **a** females **b** males. Symbols: purple triangles = *Polistes bischoffi*, red dots = *Polistes helveticus*; closed symbols = specimens identified by genetic clustering and morphological characters; open symbols = specimens identified by morphological characters only. In parentheses the variance explained by the first shape PC.

As mentioned in the introduction, two of the main target OTUs of our study, *Polistes bischoffi* and *Polistes helveticus*, are separated from the others by a reduced epicnemial carina. We therefore conducted a shape PCA including only these two OTUs for examining their morphometric differences. Only the first shape PC was informative and was plotted against isosize to evaluate the amount of allometric variation in the data (Fig. 4). Both sexes were well differentiated by the first shape PC. Furthermore, females of *Polistes helveticus* were very slightly larger than those of *Polistes bischoffi* (4a), whereas males were entirely overlapping in the size range (Fig. 4b).

**Figure 5.**
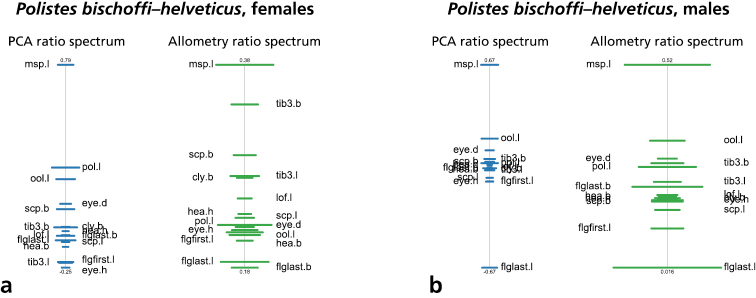
PCA ratio spectrum (blue) and allometry ratio spectrum (green) of a comparison of *Polistes bischoffi* with *Polistes helveticus*. **a** females **b** males. Horizontal bars in PCA and allometry ratio spectrum represent 68% bootstrap confidence intervals based on 1000 replicates.

To interpret the first shape PC, the PCA ratio spectrum was plotted (Fig. 5, graph with blue bars). In a PCA ratio spectrum, only ratios calculated with variables lying at the opposite ends of the spectrum are relevant for a particular shape PC (Baur and Leuenberger 2011). In a similar manner, the most allometric ratios are found in an allometry ratio spectrum (Fig. 5, graph with green bars). For females (Fig. 5a) the PCA ratio spectrum was dominated by ratios msp.l: eye.h, msp.l: tb3.l, and msp.l: flgfirst.l; for males (Fig. 5b) only a single ratio was most important, msp.l: flglast.l. The same ratio was also the most allometric (though both variables showed broad confidence intervals, see allometry ratio spectrum for males, Fig. 5b), whereas for females the dominating ratios were not among the most allometric ones (Fig. 5a). This result was in accordance with the general observation that allometric variation played a minor role in distinguishing the two groups, as they were of comparable size (compare Fig. 4).

The LDA ratio extractor is a tool for finding the best discriminating ratios for use in identification keys and diagnoses (see Baur and Leuenberger 2011). In contrast to a PCA, group membership must be specified beforehand. The results are compiled in Table 5 showing various contrasts, listed by sex. Generally, males were more distinct than females, as the groups were more widely separated in their ranges and the standard distances were on average higher, though overlapping (3.50–8.36 for males versus 4.07–6.19 for females). The ranges of two female comparisons (*biglumis*–rest, *helveticus–bischoffi*) were more or less distinct, for males a third one could be added (*bischoffi–hellenicus*). Ratios that separated the groups well were used for the key and diagnoses (see below). For both sexes, *δ* (a measure of how well shape discriminates in comparison with size) was always relatively close to zero (0.01–0.31), indicating that separation was mainly due to shape rather than size.

**Table 5.** Best ratios found by the LDA ratio extractor for separating various groupings of *Polistes*. Asterisks mark those comparisons, where the groups have very little or no overlap and the best ratios thus were eligible for use in the identification key.

**females**					
group comparison	best ratio	range group 1	range group 2	standard distance	δ
*biglumis*–rest*	msp.l: pol.l	1.22–1.76	0.79–1.24	4.62	0.29
helveticus–bischoffi*	tib3.l: msp.l	7.61–9.33	9.57–11.5	6.19	0.10
*helveticus–gallicus*	hea.b: msp.l	7.29–8.80	8.23–10.08	4.34	0.05
*helveticus–hellenicus*	tib3.l: msp.l	7.61–9.33	9.00–11.13	4.72	0.09
*bischoffi–gallicus*	flglast.l: msp.l	1.00–1.24	0.84–1.07	4.07	0.19
*bischoffi–hellenicus*	flglast.l: msp.l	1.00–1.24	0.74–1.11	4.25	0.22
**males**					
group comparison	best ratio	range group 1	range group 2	standard distance	δ
*biglumis*–rest*	lof.l: cly.b	1.29–1.45	1.46–1.69	8.36	0.31
helveticus–bischoffi*	flglast.l: msp.l	1.08–1.72	1.93–2.75	6.82	0.08
*helveticus–gallicus*	flglast.l: pol.l	0.83–1.32	1.09–1.55	3.54	0.09
*helveticus–hellenicus*	flglast.l: msp.l	1.08–1.72	1.44–2.16	5.92	0.01
*bischoffi–gallicus*	flglast.l: msp.l	1.93–2.75	1.28–2.21	3.50	0.18
bischoffi–hellenicus*	hea.b: hea.h	1.21–1.29	1.15–1.21	6.27	0.10

## Taxonomic treatment

### a) Status of OTUs

Our molecular and morphometric analyses clearly revealed that all operational taxonomic units (hitherto called OTUs) formed well-supported taxonomic units (i.e., species). We can thus confidently conclude that the three species examined in this study, *Polistes bischoffi*, *Polistes gallicus*, and *Polistes helveticus* sp. n., represent valid species.

### b) Diagnoses and descriptions of species

The following section provides information on all five species of the *gallicus*-group, as these can most easily be confused with each other, including the two main target taxa, *Polistes bischoffi* and *Polistes helveticus* sp. n.

#### Polistes biglumis

(Linnaeus, 1758)

http://species-id.net/wiki/Polistes_biglumis

Vespa biglumis Linnaeus, 1758: 573 – Holotype female (LSL), designation by Day (1979), type locality Europe (not examined)
Vespa rupestris Linnaeus, 1758: 573 – Holotype male (LSL), designation by Day (1979), type locality Sweden (not examined)
Vespa bimaculata Geoffroy in Fourcroy 1785: 433 – Holotype female (type lost; see Blüthgen 1961: 54), type locality near Paris, France
Polistes geoffroyi Lepeletier & Serville, 1825: 173 – Syntypes males, females (depository unknown), type locality France
Polistes dubius Kohl, 1898: 90 – Lectotype male (NHMW), designated by Blüthgen (1943: 128), type locality Brühl, Austria (examined by RN)
Polistes kohli Dalla Torre, 1904: 70 – Replacement name for *Polistes dubius* Kohl, 1898, nec Saussure, 1867
Polistes bimaculatus pamirensis Zirngiebl, 1955: 381–383, 385 – Syntypes 4 females (ZSM), type locality “Umss-Tugai”, probably in the area of eastern Uzbekistan to southwestern Tadjikistan (examined by RN)
Polistes bimaculatus nigrinotum Zirngiebl, 1955: 381–383, 385 – Holotype female (ZSM), type locality Althegnenberg, Germany (examined by RN)

##### Diagnosis.

Relatively large, dark species with pedicel and flagellum dorsally black in both sexes.

Females: Epicnemial carina pronounced (Fig. 12a). Hypopygium black. Metacoxa black. Mesoscutum black. Propodeum black laterally, occasionally with small yellow spot. Clypeus breadth: malar space 3.06–3.58; head breadth: malar space 6.81–8.09; malar space: lateral ocelli distance 1.22–1.76; metatibia length: malar space 6.96–8.56; terminal flagellomere length: malar space 0.64–0.87.

Males: Gena in dorsal view convex (Fig. 12l). Epicnemium and mesosternum yellow. Head breadth: head height 1.18–1.28; lower face: clypeus breadth 1.29–1.45; terminal flagellomere length: lateral ocelli distance 0.85–1.38; terminal flagellomere length: malar space 0.72–1.07; terminal flagellomere length: terminal flagellomere breadth 1.64–2.68.

##### Comments.

The holotype of *Vespa biglumis* Linnaeus, 1758, presently held at the Linnean Society of London, is not available for loan. We have, however, examined pictures online (http://linnean-online.org/16745/). Although no clear epicnemial carina is recognizable from the picture due to the condition of the specimen, the pubescence on the mesoscutum appears too long for *Polistes helveticus* sp. n. Therefore, we have no reason to question the current concept of *Polistes biglumis*.

Similarly, we have examined pictures (http://linnean-online.org/16772/) of the holotype (LINN 2807) of *Vespa rupestris* Linnaeus, 1758, also held at the Linnean Society of London and unavailable for loan. The genae of this male specimen are clearly convex in dorsal view (Fig. 12l), excluding any confusion with *Polistes helveticus* sp. n. or *Polistes bischoffi*.

The holotype of *Vespa bimaculata* Geoffroy in Fourcroy, 1785 is missing (Blüthgen 1961: 54), as are the syntypes of *Polistes geoffroyi* Lepeletier & Serville, 1825. According to the original descriptions both taxa seem to refer to dark individuals, but since no epicnemial carina is mentioned, a synonymy with *Polistes helveticus* sp. n. can neither be excluded nor proved.

The lectotype of *Polistes dubius* Kohl, 1898 was examined; we did not detect any characters allowing separation from *Polistes biglumis*. This view is also supported by our morphometric analyses (Fig. 3b, d [I]), which revealed that the lectotype of *Polistes dubius* does not plot far away from other males of *Polistes biglumis*. In any case it is a male with convex genae (Fig. 12l), making any confusion with the otherwise similarily colored male of *Polistes helveticus* sp. n. impossible.

We have seen three (ZSM-HYM-000006, ZSM-HYM-000007, ZSM-HYM-000009) of four syntypes of *Polistes bimaculatus pamirensis* Zirngiebl, 1955. Although they are dark females, occasionally with the entire mandible (ZSM-HYM-000007) or the apical part of the clypeus (ZSM-HYM-000006, ZSM-HYM-000007) black, the flagellum is not dark even on its dorsal side. The epicnemial carina is very pronounced in all three specimens, excluding confusion with *Polistes helveticus* sp. n. or *Polistes bischoffi*. However, morphology as well as morphometry (Fig. 3a, c [A]) cast doubt on whether this taxon is conspecific with *Polistes biglumis*. More material and further studies are needed to elucidate the status of this taxon.

The holotype (ZSM-HYM-000008) of *Polistes bimaculatus nigrinotum* Zirngiebl, 1955 is a very dark female; the apical part of the clypeus is entirely black and there is only a very small yellow spot on the mandible. The epicnemial carina is distinct, excluding confusion with *Polistes helveticus* sp. n. We see no trait distinguishing this specimen from *Polistes biglumis*, a view supported by our morphometric analysis (Fig. 3a, c [B]).

##### Material examined.

1 ♂ (Lectotype of *Polistes dubius*): Austria, Lower Austria, **Brühl**, 22 Aug 1883, Franz Friedrich Kohl det., NHMW coll.; 2 ♂ (RN0123, RN0124): CROATIA, ISTRIA, **Vela Učka**, 45°18'25.7"N, 14°11'40.4"E, 824 m, 27 Jul 2012, karst mountain range, Rainer Neumeyer leg., RN coll.; 1 ♂ (RN0224): GERMANY, BADEN-WÜRTTEMBERG, **Pullendorf**, 13 Jul 2009, railroad area, Mike Herrmann leg., MH coll.; 1 ♀ (Holotype of *bimaculatus nigrinotum*: ZSM-HYM-000008): Bavaria, **Althegnenberg**, 19-20 Jul 1946, Heinz Freude leg., ZSM coll.; 1 ♂ (RN0231): SWITZERLAND, CANTON BASLE-City, **Basel, Badischer Bahnhof**, 47°34'50.12"N, 07°36'07.63"E, 255 m, 18 Aug 1995, railroad area, Rainer Neumeyer leg., RN coll.; 1 ♂ (RN0247): CANTON GRISONS, **Buseno, Monti di San Carlo**, 1200 m, 09 Jul 1942, Adolf Nadig leg., ETHZ coll.; 1 ♀ (RN0249): **Davos, Züge**, 1500 m, 27 Aug 1931, Johann Peter Wolf leg., ETHZ coll.; 1 ♀ (RN0239): **Feldis/Veulden**, 21 Sep 1935, Adolf Nadig leg., ETHZ coll.; 1 ♂ (RN0248): 25 Aug 1944, Adolf Nadig leg., ETHZ coll.; 1 ♀ (RN0246): **Ftan**, 1610 m, 21 Jul 1994, Bernhard Merz leg., ETHZ coll.; 1 ♀ (RN0229): **Klosters-Serneus**, **Boschga**, 46°52'52.20"N, 09°52'14.34"E, 1060 m, 01 May 1993, montane meadow, Rainer Neumeyer leg., RN coll.; 2 ♂ (RN0250, RN0258): **Ramosch**, 07 Sep 1963, Willi Sauter leg., ETHZ coll.; 1 ♀ (RN0238): **Sumvitg**, **Rabius**, 24 Jun 1934, Adolf Nadig leg., ETHZ coll.; 1 ♂ (RN0255): **Val Müstair**, **Lü**, 13 Aug 1935, Adolf Nadig leg., ETHZ coll.; 1 ♀ (RN0028): **Val Müstair**, **Müstair: Munt Masaun**, 46°37'02.21"N, 10°25'56.36"E, 1420 m, 13 Aug 2011, rock steppe, Rainer Neumeyer leg., RN coll.; 1 ♂ (RN0027): **Val Müstair**, **Sta. Maria: Costas**, 46°36'25.78"N, 10°25'29.37"E, 1350 m, 13 Aug 2011, berm, Rainer Neumeyer leg., RN coll.; 1 ♀ (RN0029): **Val Müstair**, **Tschierv: God da Munt**, 46°37'48.79"N, 10°20'42.11"E, 1790 m, 14 Aug 2011, clear larch wood, Rainer Neumeyer leg., RN coll.; 1 ♀ + 1 ♂ (RN0021, RN0087): Hansueli Tinner leg., RN coll.; 1 ♀ (RN0243): **Zuoz**, 1800 m, 09 Sep 1966, Paul Bovey leg., ETHZ coll.; 1 ♂ (RN0233): CANTON ST. Gallen, **Pfäfers**, **Bläser Berg**, 46°57'24.76"N, 09°29'51.11"E, 1500 m, 23 Aug 1994, blowdown, Peter Duelli leg., RN coll.; 1 ♂ (RN0256): **Pfäfers**, 20 Sep 1955, Hedwig Huber leg., ETHZ coll.; 1 ♂ (RN0254): **Walenstadt, Steinbruch Engen**, 04 Sep 1997, quarry, Andreas Müller leg., ETHZ coll.; 1 ♂ (RN0232): CANTON SCHAFF-HAUSEN, **Merishausen, Chörblihalde**, 47°45'24.82"N, 08°37'16.59"E, 565 m, 25 Aug 2004, hay meadow, Rainer Neumeyer leg., RN coll.; 1 ♀ (RN0228): **Merishausen, Laadel**, 47°46'17.63"N, 08°36'25.42"E, 620 m, 16 Aug 2004, fallow meadow, Rainer Neumeyer leg., RN coll.; 1 ♀ (RN0225): CANTON Thurgovia, **Herdern**, 47°36'39.59"N, 08°54'20.39"E, 635 m, Aug 2005, Mike Herrmann leg., MH coll.; 1 ♀ (RN0240): CANTON Ticino, **Airolo**, 04 Aug 1933, Adolf Nadig leg., ETHZ coll.; 1 ♀ (RN0227): **Lavertezzo**, **Piano**, 46°15'54.34"N, 08°49'15.23"E, 589 m, 01 Jun 2012, Yannick Chittaro leg., YC coll.; 1 ♂ (RN0236): **Prato Sornico**, **Lovalt**, 46°23'20.36"N, 08°39'51.38"E, 610 m, 24 Jul 1994, riparian zones, Rainer Neumeyer leg., RN coll.; 1 ♀ (RN0178): **Prugiasco**, **San Carlo di Negrentino**, 46°27'46.14"N, 08°55'24.50"E, 860 m, 20 Aug 1993, montane meadow, Rainer Neumeyer leg., RN coll.; 1 ♀ (RN0257): CANTON VALAIS, **Ausserberg, Millachra**, 46°19'08.04"N, 07°50'14.02"E, 1210 m, 09 Jul 1998, Rainer Neumeyer leg., RN coll.; 1 ♂ (RN0234): 06 Sep 1998, Rainer Neumeyer leg., RN coll.; 1 ♀ (RN0244): **Erschmatt, Rüemetschbodu**, 46°19'35.42"N, 07°41'44.68"E, 1490 m, 18 Jul 2003, Alexandra Breitenstein leg., ETHZ coll.; 1 ♂ (RN0252): **Fiesch**, 28 Jul 1942, Adolf Nadig leg., ETHZ coll.; 1 ♂ (RN0253): **Grimentz**, 18 Aug 1941, Adolf Nadig leg., ETHZ coll.; 1 ♂ (RN0235): **Guttet-Feschel**, 1300 m, 1993, Gerhard Bächli leg., RN coll.; 1 ♀ (RN0241): **Ried-Brig**, **Berisal**, 30 Jun 1919, anon. leg., ETHZ coll.; 1 ♀ (RN0226): **Ried-Brig**, **Gantertal**, 46°17'56.61"N, 08°03'35.70"E, 1420 m, 26 Jun 2012, Yannick Chittaro leg., YC coll.; 3 ♀ (Syntypes of *bimaculatus pamirensis*: ZSM-HYM-000006, ZSM-HYM-000007, ZSM-HYM-000009): Uzbekistan or Tadjikistan, “**Umss-Tugai**”, 25 Jul 1928, Willi Rickmers leg., ZSM coll.

#### Polistes bischoffi

Weyrauch, 1937 rev. stat.

Polistes bischoffi Weyrauch, 1937: 274 – Neotype female (NMBE), present designation, type locality Galeria, Corsica, France
Polistes cf. *gallicus* – Neumeyer et al. (2011)

##### Type study.

**Figure 6.**
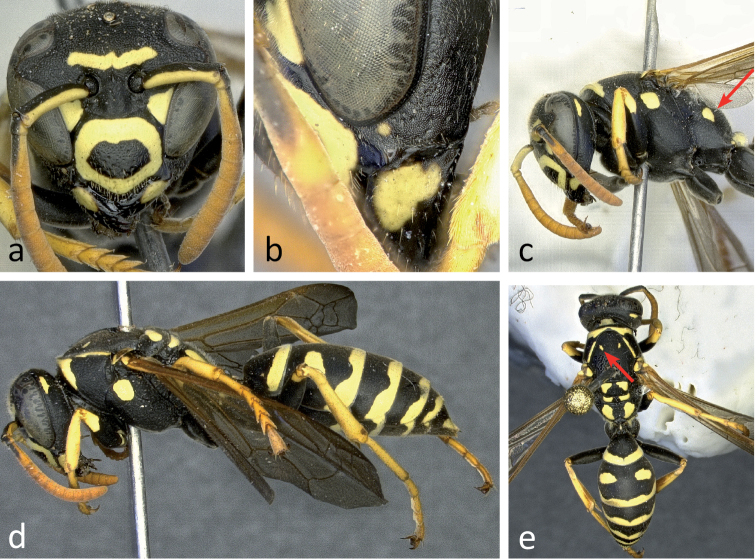
Different aspects of a female (specimen RN0137) of *Polistes bischoffi* Weyrauch, 1937: **a** frontal view of head **b** lateral view of lower face with malar space and mandible **c** lateral view of head and mesosoma **d** lateral view of body **e** body from above. Arrows indicate the yellow patch on the lateral part of propodeum (**c**) and one of two yellow spots on the mesoscutum (**e**).

*Polistes bischoffi* was described by Weyrauch (1937: 274) in a mere footnote indicating neither the type material nor the type locality. Later, Weyrauch (1938: 277 ff.) gave a key to the Palearctic species of *Polistes*, including *Polistes bischoffi*, but a more precise indication of the type material and the type locality is given only in Weyrauch (1939: 163), where a female from Macomer (Sardinia, Italy) is mentioned as the “type [Typus]”. However, following article 74.5 of the ICZN (2012) this specimen is considered as a lectotype here. Unfortunately, this lectotype is lost (Blüthgen 1956: 85), as well as most paralectotypes from various localities (Italy, Malta, and Turkey; see Weyrauch 1939: 164), with the exception of two presumed paralectotypes that we were able to examine: a female (RN0287) from the Greek Island of Poros (see below, examined material), and a male (RN0325) from Glattbrugg in Switzerland. While the male from Glattbrugg clearly belongs to the dark (Fig. 10), northern (Fig. 11) taxon (*Polistes helveticus* sp. n.), the female from Poros belongs without any doubt to the southern (Fig. 11), bright (Fig. 6) taxon (*Polistes bischoffi*). Consequently, Weyrauch (1939) most likely considered both taxa as geografically separated color morphs of the same species. Evidence for this statement can be found in his redescription of *Polistes bischoffi* (Weyrauch 1939: 163 ff.), where he writes that the antenna is “dorsally blackened in the northern part of the species range [*Fühler im Norden des Verbreitungsgebietes oberseits geschwärzt*]”.

It must be stressed that both taxa (*Polistes bischoffi*, *Polistes helveticus* sp. n.) run to “*bischoffi*” in the keys of Weyrauch (1938: 277 ff.; 1939: 195 ff.). In more recent keys (Blüthgen 1961, Dvořák and Roberts 2006, Guiglia 1972, Mauss and Treiber 2004, Witt 2009) for Central Europe however, *Polistes helveticus* sp. n. would run to “*bischoffi*”, whereas *Polistes bischoffi* would run to “*gallicus*” due to the entirely bright flagellum.

Unfortunately, the identity of the lost lectotype from Macomer (Sardinia, Italy) is unclear and can not be guessed from Weyrauch (1937, 1938, 1939). Therefore, the designation of a neotype is necessary for the clarification of the identity of *Polistes bischoffi*. Our attempts to locate the lectotype in all institutions likely to host some of Weyrauch’s material were unsuccessful (e.g.: MFNB, Michael Ohl, pers. comm.; MHNL, Claus Rasmussen, pers. comm.; FMLT, Emilia Perez, pers. comm.), and so were our attempts to locate any specimen of *Polistes bischoffi* from Sardinia, including during a field trip to Macomer in 2013. Consequently, we designate a female from Galeria on the island of Corsica (France), north of Sardinia, as the neotype of *Polistes bischoffi*. Given that there is only a distance of 12 km between the two neighboring islands (Corsica, Sardinia), and that both of them share a similar fauna (Corti et al. 1999; Kwet 2005; Tolman and Lewington 1997), we are confident that this specimen matches the lost lectotype of *Polistes bischoffi* Weyrauch, 1937. In fact both, Corsica and Sardinia are probably located too far south to host the taxon called *Polistes helveticus* sp. n. here, since the southernmost individual (RN0378) of *Polistes helveticus* sp. n. that we are familiar with was found about 200 km north of the French Mediterranean coast (Fig. 11). Moreoever, the neotype is a well preserved female of the southern, light colored species (*Polistes bischoffi*) that appears at the center of the scatter of points in our morphometric analysis and clearly lies outside the area of overlap with *Polistes gallicus* (Fig. 3a, c [C]). Lastly, this specimen (RN0366) yielded high-quality DNA and could be included in our molecular analysis.

##### Diagnosis.

**Figure 7.**
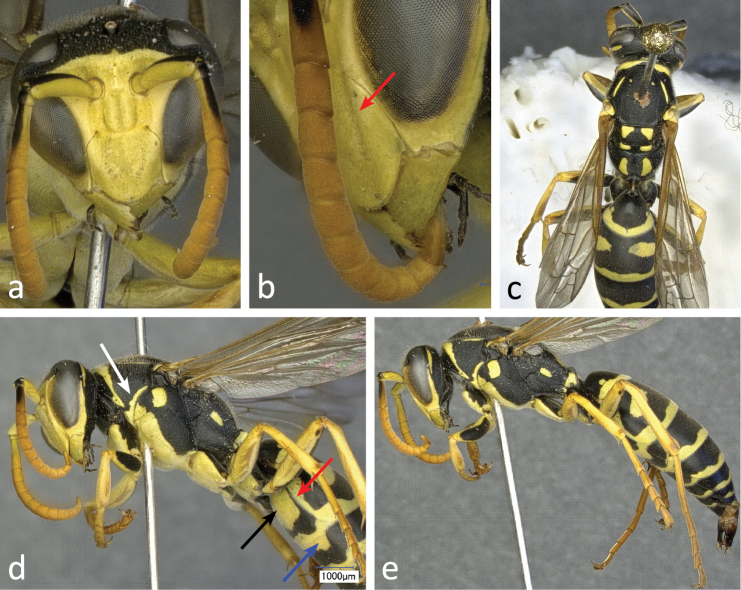
Different aspects of a male (specimen RN0151) of *Polistes bischoffi* Weyrauch, 1937: **a** frontal view of head **b** lateral view of lower face with malar space and mandible **c** dorsal view of body **d** lateral view of head, mesosoma, and base of metasoma **e** lateral view of body. The red arrow in **b** is pointing to the faint lateral ridge on clypeus. In **d** the white arrow is pointing to the yellow ventrolateral stripe of the pronotum, the red arrow to the lateral extension of terminal band on tergum 2, the black arrow to a large yellow spot on sternum 2 and the blue arrow on the yellow basal band of sternum 3.

Small and moderately bright species with flagellum on upper side bright yellow in both sexes (Figs 6a, 6c, 6d, 7a, 7b, 7d, 7e) or faintly darkened, especially in large females; pedicel and extreme base of flagellomere 1 always black on upper side.

Females: Epicnemial carina reduced (Figs 6c, 12b) or absent. Hypopygium black. Metacoxa usually black, seldom spotted yellow on upper side. Mesoscutum usually black, seldom with minute pair of yellow spots (Fig. 6e; arrow). Propodeum laterally usually with yellow spot (Fig. 6c; arrow). Clypeus breadth: malar space 3.85–4.55; head breadth: malar space 9.02–10.89; malar space: lateral ocelli distance 0.79–1.11; metatibia length: malar space 9.57–11.5; terminal flagellomere length: malar space 1.00–1.24.

Males: Gena in dorsal view immediately narrowing behind eye (Fig. 12m). Epicnemium and mesosternum yellow. Head breadth: head height 1.21–1.29; lower face: clypeus breadth 1.47–1.63; terminal flagellomere length: lateral ocelli distance 1.19–1.55; terminal flagellomere length: malar space 1.93–2.75; terminal flagellomere length: terminal flagellomere breadth 2.46–2.87.

##### Description of female.

Body length 9.9–14.1 mm (n = 22); forewing length 7.8–11.4 mm (n = 22).

Head: Clypeus yellow, with a black margin and a large central black spot usually isolated (Fig. 6a) but seldom shaped like a (rhomboid) crossband reaching lateral margin. Face with large, almost triangular yellow spot touching inner orbit (Fig. 6a). Upper gena with small, elongate spot (Figs 6c, 6d). Frons with usually uninterrupted horizontal yellow stripe (Fig. 6a).

Mesosoma: Change in sculpture between coarse mesepisternum and smooth epicnemium frequently gradual (Fig. 12b). Pronotum along posterior margin with pair of longitudinal yellow stripes not reaching yellow cross stripe on pronotal collar (Fig. 6e). Scutellum with pair of yellow, somewhat triangular spots, followed by rectangular pair of spots on metanotum and crescent-shaped pair of spots on dorsal propodeum (Fig. 6e). Mesopleuron with yellow spot (Figs 6c, 6d). Propodeal valve yellow (Fig. 6c). Tegula yellow anteriorly and posteriorly, with transparent area in between (Fig. 6e). Legs apically yellow and orange, black only on coxa, trochanter and most of femur (Figs 6d, 6e), including base.

Metasoma: Each tergum with continuous, but indented terminal yellow band (Figs 6d, 6e). Tergum 2 also with two large yellow spots (Fig. 6e). Tergum 1 occasionally with two small yellow spots. Sterna 2 and 3 with continuous terminal yellow bands, on sternum 3 occasionally centrally indented close to interruption. Sternum 4 with interrupted terminal yellow band. Sternum 5 with broadly interrupted terminal band, reduced to two lateral yellow spots.

##### Description of male.

Body length 11.3–13.4 mm (n = 8); forewing length 9.3–9.8 mm (n = 8).

Head: Mandibles, malar space, clypeus (Figs 7a, 7b), elongate spot on upper gena (Figs 7c, 7d, 7e), face and inferior frons yellow. Superior frons, vertex, occiput and back of head black (Figs 7a, 7c, 7e). Clypeus apically rounded (Fig. 7a), with faint lateral ridges extending toward orbital bays (Fig. 7b; arrow).

Mesosoma: Pronotum with yellow cross stripe along collar, often extending down both sides to longitudinal pair of yellow stripes along pronotal side margin (Fig. 7d; white arrow). Epicnemium and mesosternum yellow (Fig. 7d). Legs yellow and partially orange, except for superior side of coxa, trochanter and femur, which are black (Figs 7d, 7e). Rest of mesosoma colored as in females.

Metasoma: Tergum 2 with terminal yellow band laterally extending towards base (Fig. 7d; red arrow), even if occasionally interrupted. Terga otherwise colored as in females. Sternum 2 with pair of large yellow spots mostly isolated (Fig. 7d; black arrow), seldom fused. Sternum 3 with both terminal and basal yellow bands (Fig. 7d; blue arrow). Sterna 4 and 5 both with continuous terminal yellow band, the latter interrupted on sternum 6 and absent on hypopygium.

##### Comments.

This is one of the smallest *Polistes* species in Europe and besides *Polistes helveticus* sp. n., the only one with often absent epicnemial carina in the female sex. The two locally syntopic species (Fig. 11; Neumeyer et al. 2011) are, however, easy to distinguish in both sexes due to differently colored antennae. Furthermore, the ratio metatibia length: malar space is an unambiguous separator for females, whereas the best separating ratio for males (*Polistes bischoffi*, *Polistes helveticus* sp. n.) is the ratio terminal flagellomere length: malar space (Table 5). The same ratio weakly separates the sometimes similar females of *Polistes bischoffi* and *Polistes gallicus*. It is impossible to confuse the males of *Polistes bischoffi* with the males of *Polistes hellenicus* or *Polistes biglumis* due to the strikingly different color patterns and the diagnostic head shape of *Polistes biglumis* males within the *gallicus*-group.

Two morphs can be distinguished within *Polistes bischoffi* (rev. status), one with the flagellum entirely bright (yellow to orange) and the other with the flagellum dorsally faintly darkened. Often, the brighter morph (e.g. RN0137) has the clypeus with a central black spot (Fig. 6a), whereas in the darker morph the clypeus usually has a horizontal black band reaching the lateral margin. These two color morphs are probably the two extremes of an otherwise gradual continuum, but more individuals would have to be examined to verify this hypothesis. It would be even more important to examine whether such color variations are associated with geography or not. Limited evidence suggests that these variations are not associated with geographic location, since two nests were found (16 Aug 2013) in Zurich (Katzensee Allmend) with both morphs in each. In these colonies, the dark morph was more common among large females (presumably young queens), rather than among small females (presumably workers) or males. Also the neotype (RN0366) of *Polistes bischoffi* belongs to the darker morph and is presumably a queen, since it was collected on 19 April. More observations are needed to confirm this correlation between coloration and caste. Different color morphs within the same nest population are also reported in *Polistes gallicus* (Gusenleitner 1985: 105).

##### Distribution.

Based on the material that we have examined, *Polistes bischoffi* occurs at least in Southern Europe and Turkey from the Atlantic coast of southern France to Turkish Kurdistan (Fig. 11). The northernmost confirmed locality is in the Pannonian region of Austria (Neusiedl am See), followed by several localities in Switzerland where the species occured already in 1927 at the river Versoix near Geneva (individuals RN0170, RN0171). In all other, more northern Swiss sites *Polistes bischoffi* occurs syntopically (Neumeyer et al. 2011) with *Polistes helveticus* sp. n. and was not detected before 1992, suggesting a possible recent range expansion due to climate warming.

##### Ecology.

According to our experience in Switzerland, *Polistes bischoffi* appears to be restricted to large wetlands, especially to fens on lake shores, more so than *Polistes helveticus* sp. n. The altitudinal records range from sea level for several beach records (see “Material examined”), including the neotype (RN0366), to 540 m a.s.l. for a female (RN0076) in Switzerland (Wetzikon, Canton of Zurich). However, the Turkish locality (road from Yüksekova to Şemdinli) where three females (RN0363, RN0364, RN0365) were found was probably higher than 540 m a.s.l., since Yüksekova is situated at 1950 m, Şemdinli at 1450 m a.s.l., but the precise elevation of the locality is neither indicated on the label nor in the publication (Madl 1997: 824). Most individuals were found in August or September. The earliest record in the season is the neotype female from Galeria on 19 Apr 2002, the latest a female from a still-active nest in Mönchaltorf on 10 September 2010. The earliest male (RN0022) recorded so far was captured at Pfäffikon (Switzerland) on 10 Aug 2011, whereas the latest males (RN0082, RN0083) recorded are from Wetzikon on 09 Sep 2011. Nesting habits are apparently similar to those of *Polistes helveticus* sp. n., even where the two species were encountered syntopically (Neumeyer et al. 2011). We also found two nests in Zurich (Katzensee Allmend, 16 Aug 2013) with more than 20 and 30 individuals, respectively. These two nests were larger than any of the 14 nests described by Neumeyer et al. (2011: 13). While the smaller of both nests was attached to the dry stem of an Apiaceae, the larger one was attached to the stem of a live yellow loosestrife (*Lysimachia vulgaris*).

##### Material examined.

Neotype ♀ (RN0366): France, Corsica, **Galeria**, 42°25'11"N, 08°39'37"E, 0 m, 19 Apr 2002, estuary, Christian Monnerat leg., NMBE coll.

Paralectotype: 1 ♀ (RN0287): Greece, ATTICA, **Poros**, Moritz von Leonhardi (1856-1910) leg., SDEI coll., labeled as follows: 1. “Poros” [handwritten; misspelled as “Toros” in Weyrauch 1939: 164; see Blüthgen 1961: 56]; 2. “Coll. v. Leonhardi” [printed]”; 3. “Poliistes [sic!] ♀ gallicus L.” [handwritten, possibly from v. Leonhardi; according to Stephan Blank (pers. comm.)]; 4. “Weyrauch det. 1937.” [handwritten]; 5. “Polistula bischoffi Weyrauch” [handwritten; possibly from Weyrauch; according to Stephan Blank (pers. comm.)]”.

Further material: 1 ♀ (RN0415): Austria, Burgenland, **Neusiedl am See**, 25 Jul 1989, Michael Madl leg., NHMW coll.; 1 ♀ (RN0414): 20 Aug 1991, Michael Madl leg., NHMW coll.; 1 ♀ (RN0323): FRANCE, BOUCHES- DU-RHÔNE, **Miramas, Étang de Berre**, 15 Jul 1979, M. Kühbandner leg., MSNV coll.; 1 ♀ (RN0380): **Saintes-Maries-de-la-Mer, Camargue**, 28 Jul 2002, J. & I. Smit leg., JS coll.; 1 ♀ (RN0381): Hérault, **Vendres**, 43°13'00"N, 03°14'38"E, 0 m, 29 Jul 2009, beach, J. & I. Smit leg., JS coll.; 2 ♀ (RN0382, RN0383): **Palavas-les-Flots**, 04 Jul 2005, dunes, J. & I. Smit leg., JS coll.; 1 ♀ (RN0379): Landes, **Vielle-Saint-Girons**, **Huchet**, 04 Jul 2006, dunes, J. & I. Smit leg., JS coll.; 2 ♀ (RN0367, RN0368): Var, **Fréjus, Saint-Aygulf**, Jul 1924, Ferrière leg., NMBE coll.; 1 ♀ (RN0385): **Roquebrune-sur-Argens**, 14 Jul 2001, J. & I. Smit leg., JS coll.; 1 ♀ (RN0384): road (D560) from **Saint-Maximin-la-Sainte-Baume** to **Nans-les-Pins**, 350 m, 15 Jul 2001, J. & I. Smit leg., JS coll.; 1 ♀ (RN0370): Vaucluse, **Villelaure**, 18 Jul 2000, Jan Smit leg., JS coll.; 1 ♀ (RN0391): GREECE, Achaea, **Kalogria**, 01 Jul 2007, spit, Werner Arens leg., WA coll.; 1 ♀ (RN0390): Arcadia, **Mantineia** (archaeological site), 12 Jul 1997, Werner Arens leg., WA coll.; 1 ♀ (RN0389): 06 Jul 2007, Werner Arens leg., WA coll.; 2 ♀ (RN0372, RN0373): Euboea, **Chalkida, Camping Paradiso**, 15 Jul 1982, M. & G. Osella leg., MCSNV coll.; 2 ♀ (RN0392, RN0393): Laconia, **Chosiari, Vathi**, 09 Jun 1998, beach, Werner Arens leg., WA coll.; 1 ♀ (RN0322): ITALY, LAZIO, **Roma, Torrimpietra**, 10 Aug 1971, Heiss leg., MSNV coll.; 1 ♀ (RN0410): **Roma**, Sep 1942, O. Querci, MSNM coll.; 1 ♀ (RN0411): Lombardia, **Guardamiglio, Fiume Po**, 12 Aug 1974, river bank, Vincenzo Ferri leg., MSNM coll.; 1 ♀ (RN0409): Piemonte, **Cameri, Cascina Galdina**, 11 Jul 1981, glade, Vincenzo Ferri leg., MSNM coll.; 1 ♀ (RN0371): **Lombardore**, Sep 1972, Osella leg., MCSNV coll.; 2 ♀ (RN0170, RN0171): SWITZERLAND, CANTON GENEVA, **Versoix**, “**vers la Versoix**”, 1 Jul 1927, anon. leg., MHNG coll.; 1 ♀ (RN0156): CANTON VAUD, **Chabrey, La Morette**, 8 Sep 1992, fen, Richard Vernier leg., MHNN coll.; 4 ♀ (RN0135, RN0136, RN0141, RN0148): CANTON ZURICH, **Greifensee, Böschen:** 47°22'21.46"N, 08°40'03.38"E, 436 m, 11 Aug 2010, fen rotation fallow, Rainer Neumeyer leg., MZL coll.; 1 ♀ + 1 ♂ (RN0147, RN0150): 47°22'21.46"N, 08°40'03.38"E, 436 m, 11 Aug 2010, fen rotation fallow, Rainer Neumeyer leg., AMNH coll.; 1 ♀ (RN0146): 47°22'20.36"N, 08°40'02.49"E, 436 m, 11 Aug 2010, fen rotation fallow, Rainer Neumeyer leg., CM coll.; 1 ♀ (RN0142): 47°22'20.59"N, 08°40'02.64"E, 436 m, 11 Aug 2010, fen rotation fallow, Rainer Neumeyer leg., MH coll.; 1 ♀ (RN0137) + 1 ♂ (RN0151): **Mönchaltorf, Seewisen**, 47°19'17.08"N, 08°41'56.05"E, 436 m, 11 Aug 2010, fen rotation fallow, Rainer Neumeyer leg., NMBE coll.; 1 ♀ (RN0140): 47°19'17.80"N, 08°41'54.97"E, 436 m, 11 Aug 2010, fen rotation fallow, Rainer Neumeyer leg., CSE coll.; 1 ♀ (RN0001): **Pfäffikon, Auslikon**, 47°20'47.75"N, 08°47'50.16"E, 539 m, 10 May 2011, fen, Rainer Neumeyer leg., RN coll.; 1 ♂ (RN0022): **Pfäffikon, Birchen**, 47°21'03.19"N, 08°47'31.73"E, 538 m, 10 Aug 2011, fen, Rainer Neumeyer leg., RN coll.; 1 ♀ (RN0169): **Regensdorf, Altburg**, 24 Jul 1997, hill near fen, Bernhard Merz leg., MHNG coll.; 1 ♀ (RN0145): **Schwerzenbach, Böschen**, 47°22'21.48"N, 08°40'01.19"E, 436 m, 11 Aug 2010, fen rotation fallow, Rainer Neumeyer leg., NML coll.; 1 ♀ (RN0149): 47°22'21.35"N, 08°40'00.85"E, 436 m, 11 Aug 2010, fen rotation fallow, Rainer Neumeyer leg., NHMB coll.; 1 ♀ (RN0143): 47°22'21.39"N, 08°40'00.75"E, 436 m, 11 Aug 2010, fen rotation fallow, Rainer Neumeyer leg., ETHZ coll.; 1 ♀ (RN0144): 47°22'21.39"N, 08°40'00.90"E, 436 m, 11 Aug 2010, fen rotation fallow, Rainer Neumeyer leg., MCSNL coll.; 1 ♀ (RN0076): **Wetzikon, Himmerich:** 47°20'07.85"N, 08°47'31.05"E, 540 m, 02 Sep 2011, fen, Rainer Neumeyer leg., RN coll.; 1 ♀ (RN0105): **Wetzikon, Robenhuserriet**, 47°20'11.31"N, 08°47'14.84"E, 538 m, 17 Aug 2012, fen, Rainer Neumeyer leg., BNM coll.; 1 ♂ (RN0075): **Wetzikon, Seeriet**, 47°20'30.24"N, 08°47'10.89"E, 538 m, 02 Sep 2011, fen, Rainer Neumeyer leg., RN coll.; 2 ♂ (RN0082, RN0083): 47°20'30.24"N, 08°47'10.89"E, 538 m, 09 Sep 2011, fen, Rainer Neumeyer leg., RN coll.; 1 ♀ (RN0077): 47°20'29.54"N, 08°47'23.73"E, 537 m, 02 Sep 2011, fen, Rainer Neumeyer leg., RN coll.; 1 ♂ (RN0328): **Zürich, Katzensee Allmend**, 47°25'53.05"N, 08°30'26.15"E, 438 m, 16 Aug 2013, fen, André Rey leg., AR coll.; 4 ♀ (RN0329, RN0330, RN0331, RN0332) + 3 ♂ (RN0333, RN0335, RN0336): 19 Aug 2013, Rainer Neumeyer leg., RN coll.; 1 ♂ (RN0334): 19 Aug 2013, Rainer Neumeyer leg., NHM coll.; 2 ♀ (RN0337, RN0338) + 2 ♂ (RN0339, RN0340): 47°25'56.74"N, 08°30'22.41"E, 438 m, 19 Aug 2013, fen, Rainer Neumeyer leg., RN coll.; 3 ♀ (RN0363, RN0364, RN0365): Turkey, Hekarî, road from **Yüksekova** to **Şemdinli** [“Sendili”], 1450–1950 m, marshy meadow [*Sumpfwiese*], 05 Jun 1971, Michael Madl leg., NHMW coll.

#### Polistes gallicus

(Linnaeus, 1767)

http://species-id.net/wiki/Polistes_gallicus

Vespa gallica Linnaeus, 1767: 949 – Holotype male (LSL), type locality “Europa australi” [South of Europe] (not examined)
Polistes foederatus Kohl, 1898: 90 – Lectotype male (NHMW), designated by Blüthgen (1943: 129), type locality Göygöl [“Transkauk., Helenendorf”], Azerbaijan (examined by RN)
Polistes gallicus mongolicus Buysson, 1911: 218 – Syntypes males females (MNHN, ZIN), type locality road from Kuqa [“Koutchar”] to Karashahr [“Karachar”], China (Xinjiang autonomous region) (syntype male in MHNG examined by RN)
Polistes foederatus obscuricornis Mader, 1936: 263 – Syntypes females (NHMW), type locality (island of) Krk, Croatia (2 syntypes examined by RN)
Polistula omissa Weyrauch, 1938: 277 – Lectotype male (lost; see Arens 2011: 462), designated by Weyrauch 1939: 161, type locality Marseille, France, mentioned in Weyrauch (1939): 161.
Polistes omissus ordubadensis Zirngiebl, 1955: 381 – Holotype female (ZSM), type locality Ordubad, Azerbaijan (examined by RN)
Polistes omissus kaszabi Giordani Soika, 1970: 327–328 – Holotype female (HNHM), type locality “Duusch ul” near Züünkharaa [„Zuun-Chara“], Mongolia (examined by RN)
Polistes foederatus albellus Giordani Soika, 1976: 272 – Holotype female (HNHM, currently loaned elsewhere), type locality Bulgan aimag: Namnan ul mountains, 23 km NW of Somon Chutag, Mongolia (1 paratype female in MSNV examined by RN)

##### Diagnosis.

Relatively small and bright species with flagellum bright yellow to orange on upper side in both sexes; pedicel and extreme base of flagellomere 1 always black on upper side.

Females: Epicnemial carina distinct or reduced. Hypopygium black. Metacoxa frequently spotted yellow on upper side. Mesoscutum often with pair of yellow spots. Propodeum laterally with yellow spot on each side. Clypeus breadth: malar space 3.49–4.25; head breadth: malar space 8.23–10.08; malar space: lateral ocelli distance 0.87–1.2; metatibia length: malar space 8.26–11.88; terminal flagellomere length: malar space 0.84–1.07.

Males: Gena in dorsal view immediately narrowing behind eye. Epicnemium and mesosternum yellow. Head breadth: head height 1.17–1.27; lower face: clypeus breadth 1.46–1.6; terminal flagellomere length: lateral ocelli distance 1.09–1.55; terminal flagellomere length: malar space 1.28–2.21; terminal flagellomere length: terminal flagellomer breadth 1.84–3.2.

##### Comments.

The holotype of *Vespa gallica* Linnaeus, 1767 (LINN 2790), presently held at the Linnean Society of London, is not available for loan. We have, however, examined pictures (http://linnean-online.org/16757/). They clearly show the bright flagellum all around, excluding identity with *Polistes helveticus* sp. n. A careful examination of this specimen would be needed to confirm the identify of *Polistes gallicus*.

Except for *Vespa gallica* and for the lost lectotype of *Polistula omissa* Weyrauch, 1938 we have examined and measured type specimens of all taxa listed as synonymous with *Polistes gallicus*. Since each of them appears to be clearly distinct from *Polistes bischoffi* in our morphometric analyses (Fig. 3), we only compare them with *Polistes helveticus* sp. n. in the following section.

Blüthgen (1941: 245) and Guiglia (1972: 49) claim that the upper side of the flagellum of *Polistes foederatus* Kohl, 1898 is “slightly blackened [*leicht geschwärzt*]” or “darkened [*assombrie*]”, respectively, even in the male sex, unlike the flagellum of *Polistes omissus* (Weyrauch, 1938). However, the flagellum of the male lectotype of *Polistes foederatus* from Azerbaijan is bright yellow all around, as noted by Blüthgen (1943: 129), thus excluding any confusion with *Polistes helveticus* sp. n. Furthermore, the clypeus of this lectotype has not even a trace of a longitudinal furrow, although this trait has also been regarded as diagnostic for *Polistes foederatus* (Blüthgen 1941: 245). Currently, both *Polistes foederatus* and *Polistes omissus* are synonyms of *Polistes gallicus* (Day 1979: 63; Gusenleitner 1985: 105). This view is supported by our morphometric analysis (Fig. 3b, d [J]) for *Polistes foederatus*.

The lectotype of *Polistes gallicus mongolicus* Buysson, 1911 has its epicnemium and mesosternum largely black as in *Polistes hellenicus*, but is otherwise a large, very light colored male with an extremely broad head. Its flagellum is bright all around, excluding any synonymy with *Polistes helveticus* sp. n. The terminal flagellomere is in fact “very short” [*très court*], as Buysson (1911: 218) states. Morphologically it appears doubtful that the taxon *Polistes mongolicus* belongs to *Polistes gallicus* (C. van Achterberg, pers. comm.). This view is also supported by our morphometric analyses (Fig. 3b, d [K]).

We have seen two syntypes (RN0444, RN0445) of *Polistes foederatus obscuricornis* Mader, 1936. In contrast to the statement of Mader (1936: 263) the flagella of these two females are not “entirely black [*ganz schwarz*]” dorsally, but only grey. Only the scape, the pedicel, and the very base of the first flagellomere are entirely black dorsally. Both of these otherwise light colored individuals have a pronounced epicnemial carina, excluding confusion with *Polistes helveticus* sp. n. Although this taxon belongs without any doubt to the *gallicus*-group, our morphometric analyses (Fig. 3a, c [D]) do not entirely support their synonymy with *Polistes gallicus*.

The female holotype of *Polistes omissus ordubadensis* Zirngiebl, 1955 is colored very light with the clypeus unspotted, the flagellum colored light dorsally, the hypopygium (Blüthgen 1956: 85) and even the malar space largely yellow, and the epicnemial carina distinct, all of them excluding confusion with *Polistes helveticus* sp. n. This view is also supported by morphometry (Fig. 3a, c [E]). In our opinion this taxon (*Polistes omissus ordubadensis*) may not even belong to the *gallicus*-group.

The holotype of *Polistes omissus kaszabi* Giordani Soika, 1970 is a large, dark female with both malar space and mandibles entirely black. The epicnemial carina is distinct, excluding confusion with *Polistes helveticus* sp. n. Flagellum, clypeus and hypopygium are colored and patterned as in *Polistes biglumis* or *Polistes nimpha*. In fact, a synonymy with *Polistes gallicus* is not supported by our morphometric analyses (Fig. 3a, c [F]) and this taxon (*Polistes omissus kaszabi*) may not even belong to the *gallicus*-group (C. van Achterberg, pers. comm.).

The holotype of *Polistes foederatus albellus* Giordani Soika, 1976 is currently on loan and could not be examined, but we have examined the only paratype (RN0326, MSNV-04702) mentioned in Giordani Soika (1976: 272). It is an extremely dark female with the flagellum dorsally black and the epicnemial carina unilaterally reduced. It is morphologically and even morphometrically (Fig. 3a, c [G]) similar to a very dark *Polistes helveticus* sp. n., except that the bright spots and stripes are not only reduced, but also of ivory-white rather than of yellow color. These striking characters make it unlikely that this taxon (*Polistes albellus*) belongs to a species reaching Europe (C. van Achterberg, pers. comm.).

These observations indicate that the taxon *Polistes gallicus* should be carefully revised; our analysis of the mitochondrial marker further indicates that two specimens (*Polistes* sp. aff. *gallicus*; RN0126, RN0129) from Greece, both identified as *Polistes gallicus* by Arens (2011), may represent another species. The upper side of the flagellum in both of these individuals is slightly darkened. This trait, as well as others (e.g. a broad malar space), suggest that this taxon (*Polistes* sp. aff. *gallicus*) may be *Polistes foederatus obscuricornis*. More data are required to resolve this problem.

In addition, our morphometric analysis reveals considerable intraspecific heterogeneity within *Polistes gallicus*. Specimens from continental Europe (Croatia, Portugal, Switzerland) were quite different from those from Sardinia. Both taxa overlapped but exhibited considerable differences. However, our molecular analyses did not indicate any difference between continental and Sardinian specimens. Therefore, we must also consider some unknown external factors such as temperature or humidity to be at least partially responsible for the observed morphometric variation. Such factors have previously been implicated in color pattern variation (Zimmermann 1931).

##### Material examined.

1 ♂ (Lectotype of *Polistes foederatus*): Azerbaijan, Goygol District, **Göygöl** [“Transkauk., Helenendorf”], 1886, Franz Friedrich Kohl det., NHMW coll.; 1 ♀ (Holotype of *Polistes omissus ordubadensis*: ZSM-HYM-000005): Nakhchivan Autonomous Republic, **Ordubad**, 1913, Kulzer leg., ZSM coll.; 1 ♂ (Lectotype of *Polistes gallicus mongolicus*; EY8898): China, Xinjiang, **road from Kuqa [“Koutchar”] to Karashahr [“Karachar”] in Kashgar prefecture [“Kachgarie”]**, 1909, L. Vaillant leg., MNHN coll.; 1 ♀ (RN0099): Croatia, Istria, **Rovinj, Cesta za Mondelaco**, 45°05'57.6"N, 13°38'36.8"E, 15 m, 24 Jul 2012, fallow, Rainer Neumeyer leg., CSE coll.; 1 ♀ (RN0100): 28 Jul 2012, fallow, Rainer Neumeyer leg., CSE coll.; 3 ♀ (RN0194, RN0195, RN0196): Rainer Neumeyer leg., RN coll.; 1 ♀ (RN0009): **Rovinj, Farma Haber**, 45°06'40.8"N, 13°40'21.0"E, 30 m, 22 Jul 2011, farm, Rainer Neumeyer leg., RN coll.; 1 ♀ (RN0197): **Vodnjan, street D21**, 44°57'58.0"N, 13°50'47.4"E, 133 m, 24 Jul 2012, fallow, Rainer Neumeyer leg., RN coll.; 2 ♀ (Syntypes of *Polistes foederatus obscuricornis*; RN0444, RN0445): Krk, ≤ 1936, NHMW coll.; 1 ♀ (RN0343): Italy, Sardinia, **Cabras, Stagno di Cabras**, 39°57'07.6"N, 08°31'17.2"E, 5 m, 31 Aug 2013, paddy, Rainer Neumeyer leg., RN coll.; 1 ♂ (RN0347): **Strada Provinciale 1**, 39°55'14.1"N, 08°31'17.4"E, 6 m, 01 Sep 2013, reed, Rainer Neumeyer leg., RN coll.; 1 ♀ (RN0344): **Macomer, Via Alagon**, 40°15'49.9"N, 08°46'54.5"E, 553 m, 01 Sep 2013, rock face, Rainer Neumeyer leg., RN coll.; 1 ♀ (RN0345) + 1 ♂ (RN0348): **Scano di Montiferro, Street SP78**, 40°13'19.9"N, 08°36'29.9"E, 442 m, 01 Sep 2013, quarry, Rainer Neumeyer leg., NHM coll.; 1 ♂ (RN0346): **Tadasuni, Street to San Serafino**, 40°05'41.4"N, 08°52'44.5"E, 138 m, 30 Aug 2013, edge community, Rainer Neumeyer leg., RN coll.; 1 ♀ (Paratype of *Polistes foederatus albellus*; RN0326, MSNV-04702): Mongolia, Bulgan aimag, **Namnan ul mountains**, 1150 m, 21 Jul 1968, Zoltán Kaszab leg., Antonio Giordani Soika det., MSNV coll.; 1 ♀ (Holotype of *Polistes omissus kaszabi*; HNHM-283): Selenge aimag, “**Duusch ul**” near **Züünkharaa [„Zuun-Chara**“], 1100 m, 08 Jul 1964, Zoltán Kaszab leg., HNHM coll.; 1 ♀ (RN0108): Portugal, Algarve, **Vila do Bispo, western shore**, 37°06'57.2"N, 08°55'37.2"W, 11 m, 04 Apr 2012, dune, Rainer Neumeyer leg., RN coll.; 1 ♀ (RN0395) + 1 ♂ (RN0396): Slovakia, Nitra Region, **Štúrovo**, 16 Sep 1947, Augustin Hoffer leg., LD coll.; 1 ♂ (RN0172): SWITZERLAND, CANTON Geneva, **Genève**,< 1900, anon. leg., MHNG coll.; 1 ♂ (RN0208): CANTON Grisons, **Roveredo**, 18 Aug 1924, Adolf Nadig leg., ETHZ coll.; 3 ♂ (RN0205, RN0206, RN0207): 17 Aug 1949, Adolf Nadig leg., ETHZ coll.; 1 ♀ (RN0118): **San Vittore, Ai Tecc**, 46°14'10.42"N, 09°05'20.18"E, 262 m, 30 Jul 2012, industrial fallow, Rainer Neumeyer leg., RN coll.; 1 ♂ (RN0166): **Savognin**, Jul 1907, anon. leg., BNM coll.; 1 ♀ (RN0165): **Val Müstair, Sta. Maria**, 20 Jul 1951, Jacques de Beaumont leg., BNM coll.; 10 ♂ (RN0173, RN0209, RN0210, RN0211, RN0212, RN0213, RN0214, RN0215, RN0216, RN0217): CANTON Ticino, **Locarno**, 05 Jul 1907, anon. leg., ETHZ coll.; 3 ♀ (RN0110, RN0111, RN0112): **Sant’ Antonino, Via Gorelle/Canvera**, 46°09'19.72"N, 08°58'18.65"E, 215 m, 30 Jul 2012, industrial fallow, Rainer Neumeyer leg., RN coll.; 4 ♀ (RN0103, RN0106, RN0107, RN0109): 08 Aug 2012, industrial fallow, Rainer Neumeyer leg., RN coll.; 2 ♀ (RN0041, RN0042): CANTON VALAIS, **Leuk, Satellitenbodenstation**, 46°19'06.66"N, 07°38'36.56"E, 919 m, 22 Aug 2011, tall herbaceous vegetation, Rainer Neumeyer leg., RN coll.; 2 ♀ (RN0104, RN0113): 07 Aug 2012, Rainer Neumeyer leg., RN coll.; 1 ♀ + 2 ♂ (RN0115, RN0116, RN0117): CANTON Vaud, **Villars-sous-Yens, Arborex**, 46°30'11.03"N, 06°25'12.11"E, 510 m, 25 Aug 2010, Christian Monnerat leg., MHNN coll.

#### Polistes hellenicus

Arens, 2011

http://species-id.net/wiki/Polistes_hellenicus

Polistes hellenicus Arens, 2011: 464 – Holotype male (WA), type locality Ano Kotili, Greece (examined by RN)

##### Diagnosis.

Relatively small, bright species with flagellum bright yellow on upper side in both sexes; pedicel and extreme base of flagellomere 1 always black on upper side.

Females: Epicnemial carina distinct. Hypopygium often orange and yellow at the tip. Metacoxa spotted yellow on upper side. Mesoscutum sometimes with pair of yellow spots. Propodeum laterally often with yellow spot on each side. Clypeus breadth: malar space 3.46–4.46; head breadth: malar space 7.75–10.65; malar space: lateral ocelli distance 0.85–1.24; metatibia length: malar space 9.00–11.13; terminal flagellomere length: malar space 0.74–1.11.

**Figure 8.**
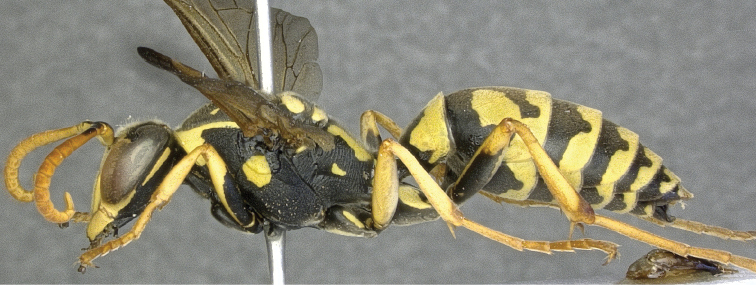
Lateral aspect of the holotype (individual RN0242; male) of *Polistes hellenicus* Arens, 2011. Unique for males of European *Polistes* is the black ventral side of the mesosoma, especially epicnemium and mesosternum.

Males: Gena in dorsal view immediately narrowing behind eye. Epicnemium and mesosternum black (Fig. 8). Head breadth: head height 1.15–1.21; lower face: clypeus breadth 1.48–1.62; terminal flagellomere length: lateral ocelli distance 1.21–1.4; terminal flagellomere length: malar space 1.44–2.16; terminal flagellomere length: terminal flagellomere breadth 2.12–2.76.

##### Comments.

The male holotype of *Polistes hellenicus* can be easily distinguished from *Polistes bischoffi* and *Polistes helveticus* sp. n. based on its black epicnemium and mesosternum (Fig. 8). From a morphometric point of view the holotype (*Polistes hellenicus*) lies at the border with *Polistes bischoffi*, but clearly outside the range of *Polistes helveticus* sp. n. (Fig. 3b, c [L]).

##### Material examined.

Holotype ♂ (RN0242): Greece, Arcadia, **Ano Kotili, peak of Lykaion**, 1100–1400 m, 07 Jul 2010, Werner Arens leg., WA coll.

Paratypes: 1 ♀ (RN0174): Greece, Arcadia, **Andritsena**, 1997, Werner Arens leg., ZSM coll.; 1 ♂ (RN0125): **Ano Kotili, peak of Lykaion**, 1100–1400 m, 07 Jul 2010, Werner Arens leg., WA coll.; 1 ♂ (RN0179): Argolis, **Drepano**, 06 Jul 2008, Werner Arens leg., WA coll.

Further material: 4 ♂ (RN0220, RN0221, RN0222, RN0223): Croatia, Istria, **Ližnjan**, 44°49’N, 13°59’E, 27 Aug 2005, Christian Schmid-Egger leg., CSE coll.; 2 ♀ (RN0008, RN0088): **Rovinj**, 45°06'07.1"N, 13°39'08.4"E, 22 m, 22 Jul 2011, Rainer Neumeyer leg., RN coll.; 1 ♀ (RN0097): **Rovinj**, **Cesta za Mondelaco**, 45°05'57.6"N, 13°38'36.8"E, 15 m, 24 Jul 2012, Rainer Neumeyer leg., CSE coll.; 1 ♀ (RN0098): 28 Jul 2012, Rainer Neumeyer leg., CSE coll.; 1 ♀ (RN0193): Rainer Neumeyer leg., RN coll.; 2 ♀ (RN0010, RN0011): **Rovinj**, **Cesta za Valaltu-Lim**, 45°06'16.8"N, 13°38'28.4"E, 26 m, 26 Jul 2011, Rainer Neumeyer leg., RN coll.; 1 ♀ (RN0191): 21 Jul 2012, Rainer Neumeyer leg., RN coll.; 1 ♀ (RN0193): **Rovinj**, **near Valalta**, 45°07'02.1"N, 13°37'54.0"E, 14 m, 21 Jul 2012, Rainer Neumeyer leg., RN coll.; 2 ♂ (RN0101, RN0102): **Vela Učka**, 45°18'25.7"N, 14°11'40.4"E, 824 m, 27 Jul 2012, karst mountain range, Rainer Neumeyer leg., RN coll.; 1 ♀ (RN0183): Greece, Arcadia, **Andritsena**, 31 May 2011, Werner Arens leg., WA coll.; 1 ♀ (RN0132): **Mantinea**, 08 Jun 2011, Werner Arens leg., WA coll.; 1 ♀ (RN0181): Elis, **Olympia**, 29 May 2011, Werner Arens leg., WA coll.; 1 ♀ (RN0131): 08 Jun 2011, Werner Arens leg., WA coll.; 1 ♀ (RN0182): **Zacharo, Lake Kaiapha**, 30 May 2011, Werner Arens leg., WA coll.; 1 ♀ (RN0096): **Zacharo, Neochori**, 30 May 2011, Werner Arens leg., RN coll.; 1 ♀ (RN0175): Ionian Islands, **Cephalonia**, 03 Sep 1992, Peter Hartmann leg., ZSM coll.; 1 ♀ (RN0127): Messenia, **Avia near Kalamata**, 01 Jun 2011, Werner Arens leg., WA coll.; 3 ♀ (RN0133, RN0185, RN0186): 02 Jun 2011, Werner Arens leg., WA coll.; 1 ♀ (RN0176): **Kalamata**, 1997, Werner Arens leg., ZSM coll.; 1 ♀ (RN0184): **Mavromati, Ithome mountain**, 01 Jun 2011, Werner Arens leg., WA coll.; 1 ♀ (RN0177): 1997, Werner Arens leg., ZSM coll.

#### Polistes helveticus

Neumeyer sp. n.

http://zoobank.org/2BF81BF0-5EB0-4C74-87C4-50A294C330A3

http://species-id.net/wiki/Polistes_helveticus

Polistes bischoffi Weyrauch, 1937: 274, in part – Weyrauch (1939), in part (paralectotype male, RN0325, of *Polistes bischoffi*, HUMCZ coll., Glattbrugg near Zurich, Switzerland). The following references published under the name of *Polistes bischoffi* Weyrauch, 1937 actually belong to *Polistes helveticus* sp. n.: Baugnée (1996), Blüthgen (1961), Dvořák and Roberts (2006), Dvořák et al. (2006), Graf (1961), Guiglia (1967, 1972), Kofler (2005), Mauss (2001), Mauss and Treiber (2004), Neumeyer et al. (2011), Schmid-Egger and Treiber (1989), Schneider et al. (1998), Witt (2009).
Polistes helveticus Holotype female (NMBE coll.), present designation, type locality Schwerzenbach, canton of Zürich, Switzerland

##### Diagnosis.

**Figure 9.**
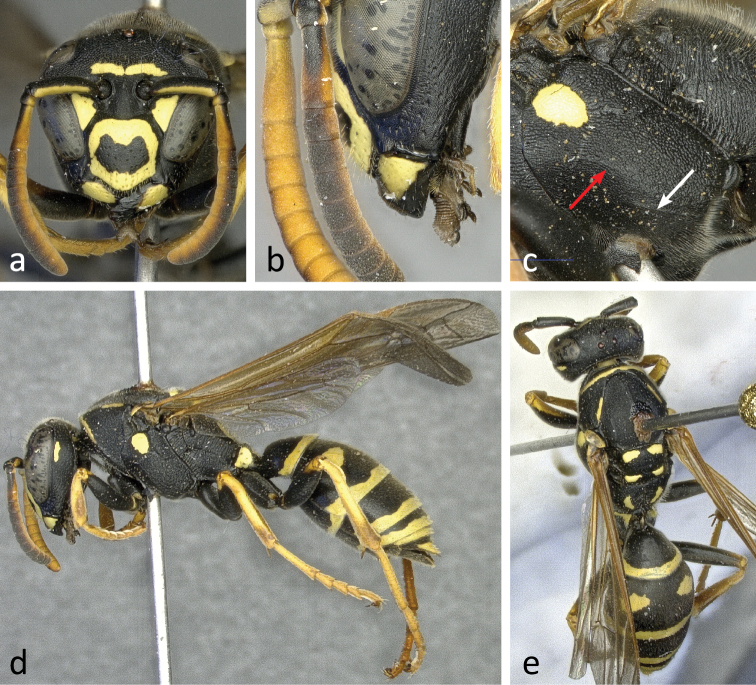
Different aspects of a female (individual RN0138) of *Polistes helveticus* sp. n.: **a** frontal view of head **b** lateral view of lower face with malar space and mandible **c** mesopleural region of mesosoma **d** lateral view of body **e** dorsal view of body. The red arrow in picture (**c**) is pointing to the rather reduced epicnemial carina, and the white arrow to the quite distinct mesopleural signum (sensu Carpenter 1996a), a structure also called a sternopleural groove (Richards 1973).

**Figure 10.**
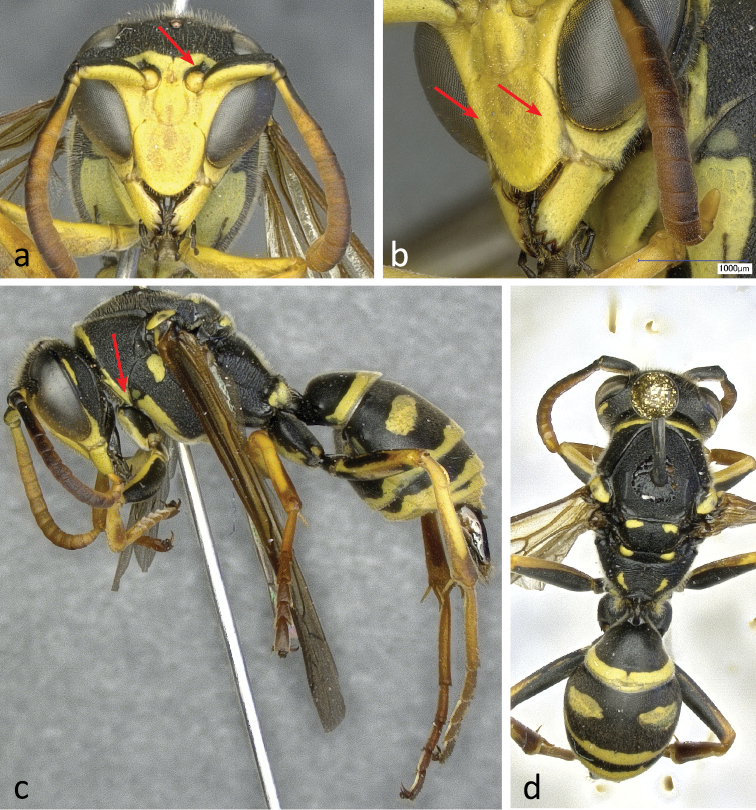
Different aspects of a male (individual RN0153) of *Polistes helveticus* sp. n.: **a** frontal view of head **b** anterolateral view of lower face **c** lateral view of body **d** dorsal view of body. The arrows are pointing to the isolated black area (**a**) bordering the torulus, the very faint lateral ridges of clypeus (**b**) or the ventrolateral angle of the pronotum (**c**).

Small and relatively dark species with pedicel and flagellum black on upper side in both sexes (Figs 9a, 9b, 10a, 10c).

Females: Epicnemial carina reduced (Fig. 9c; red arrow) or absent. Hypopygium black (Fig. 9d). Metacoxa black. Mesoscutum black (Fig. 9e), only rarely with a minute pair of yellow spots. Propodeum laterally usually black (Fig. 9d), seldom with yellow spot on each side. Clypeus breadth: malar space 3.26–3.73; head breadth: malar space 7.29–8.8; malar space: lateral ocelli distance 0.87–1.19; metatibia length: malar space 7.61–9.33. terminal flagellomere length: malar space 0.81–1.07.

Males: Gena in dorsal view immediately narrowing behind eye (Fig. 12m). Epicnemium and mesosternum yellow (Figs 10a, 10c). Head breadth: head height 1.19–1.27; lower face: clypeus breadth 1.46–1.69; terminal flagellomere length: lateral ocelli distance 0.83–1.32; terminal flagellomere length: malar space 1.08–1.72; terminal flagellomere length: terminal flagellomere breadth 1.70–2.78.

**Figure 11.**
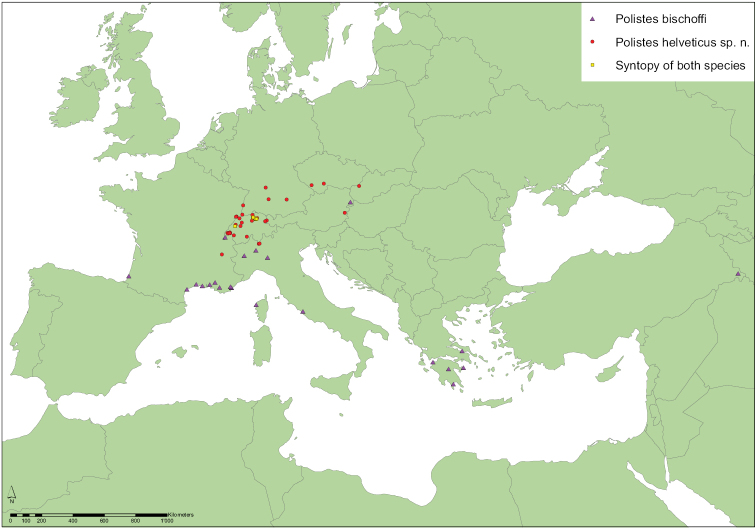
Distribution of examined specimens of *Polistes bischoffi* Weyrauch, 1937 and *Polistes helveticus* sp. n. While *Polistes bischoffi* mainly occurs from Southern Europe to Western Asia, *Polistes helveticus* appears to have a more northern distribution in Central Europe. Thus far, the only incidences of syntopy (*Polistes bischoffi*, *Polistes helveticus*) are from Switzerland.

**Figure 12.**
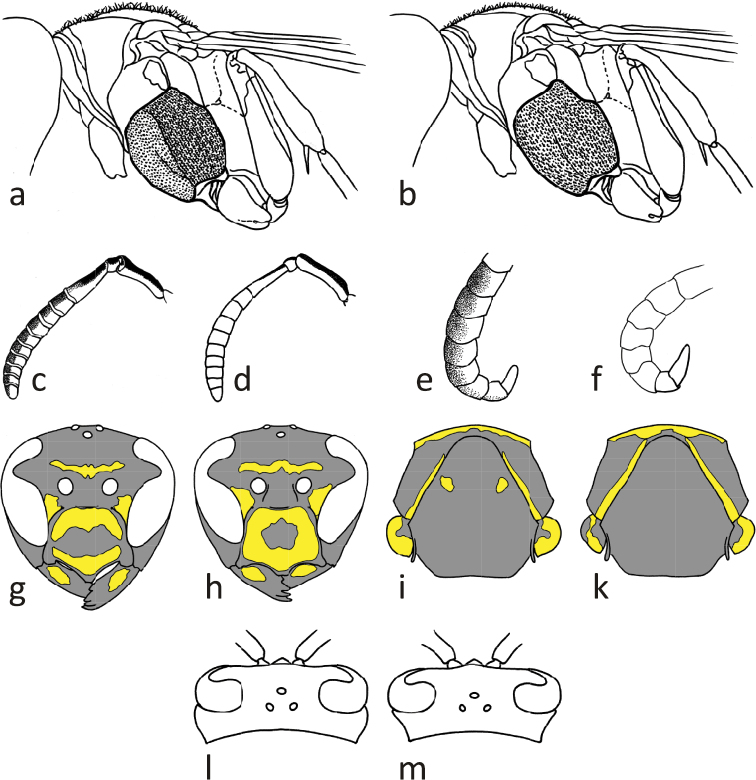
Drawings of morphological characters used in the key to European paper wasps (*Polistes*) of the *gallicus*-group: Mesopleuron with abrupt (**a**) or gradual (**b**) sculpture change; female antennae with dark (**c**) or bright (**d**) upper side of flagellomeres; male apical flagellum dark (**e**) or bright (**f**) on upper side; head in frontal view with black band across clypeus (**g**) or isolated black spot (**h**); apical mesosoma in dorsal view with drop-shaped spots on mesoscutum, posterior pronotal stripes ending far from pronotal carina (**i**) or without drop-shaped spots, pronotal stripes approaching pronotal carina (**k**); head in dorsal view with genae convex (**l**) or immediately narrowing (**m**). Drawings **a, b, c, d, f** are courtesy of H. Cigler, **g, h, i, k** of D. Lawniczak, and **e, l, m** of CSCF.

##### Description of female.

Body length 9.7–14.1 mm (n = 20); forewing length 8.5–11.3 mm (n = 20).

Head: Clypeus yellow with black margin and large central black spot; this spot either isolated (Fig. 9a) or more often extended as crossband reaching the lateral margins of clypeus (Fig. 12g). Face with nearly triangular yellow spot touching inner orbit (Fig. 9a). Upper gena with small, elongate spot (Fig. 9d). Frons with pair of horizontal yellow stripes seldom confluent (Fig. 9a).

Mesosoma: Change in sculpture between coarse mesepisternum and smooth epicnemium frequently gradual (Fig. 12b). Pronotum along posterior margin with pair of longitudinal stripes not reaching cross stripe on pronotal collar (Fig. 9e). Scutellum and metanotum each with pair of yellow bars (Fig. 9e). Propodeum dorsally usually with pair of crescent-shaped spots (Fig. 9e). Mesopleuron with yellow spot (Figs 9c, 9d). Propodeal valve yellow (Fig. 9d). Tegula yellow anteriorly and posteriorly, with more transparent area in between (Fig. 9e). Legs yellow and orange, black only on coxa, trochanter and most of femur, including entire base (Figs 9d, 9e).

Metasoma: Each tergum with continuous, but slightly indented terminal yellow band (Figs 9d, 9e). Tergum 2 also with two yellow spots (Fig. 9e). Tergum 1 seldom with two small yellow spots. Sterna 2 and 3 with terminal yellow bands usually interrupted, even though often only slightly so. Sterna 3, 4 and 5 with broadly interrupted terminal bands, manifested only as lateral terminal yellow spots.

##### Description of male.

Body length 9.6–12.4 mm (n = 12); forewing length 8.9–9.9 mm (n = 12).

Head: Mandibles, malar space, clypeus, face, inferior frons (Figs 10a, 10b) and elongate spot on upper gena (Fig. 10c) yellow. Superior frons, vertex (Fig. 10a), occiput and back of head (Figs 10c, 10d) black. Inferior part of frons yellow with small black area at superior margin of torulus, usually isolated (Fig. 10a; arrow), but seldom reaching the superior part of frons above. Clypeus apically rounded (Fig. 10a), with hardly any lateral ridge (Fig. 10b; arrows).

Mesosoma: Pronotum with yellow cross stripe along collar, occasionally extending down to sharp angle of pronotum (Fig. 10c; arrow). Legs yellow and orange, except for upper sides of coxa, trochanter and femur, which are black (Figs 10c, 10d); black area occasionally reaching (yellow) lower side of hind femur, yellow area occasionally reaching (black) upper side of pro- and mesocoxa. Rest of mesosoma colored as in females (Fig. 10d).

Metasoma: Tergum 2 with terminal yellow band extending laterally toward base, even if occasionally discontinuous. Other terga colored as in females. Sternum 2 most of the time with two yellow spots. Sterna 3, 4 and 5 usually with continuous terminal yellow band, the latter interrupted on sternum 6 and absent on hypopygium.

##### Comments.

Except for *Polistes bischoffi*, *Polistes helveticus* sp. n. is the only European species with an epicnemial carina that is often absent in the female sex. These two species are easy to distinguish in both sexes due to their differing color patterns, mainly on the antennae. Furthermore, the ratio metatibia length: malar space separates females, whereas the best separating ratio for males (*Polistes bischoffi*, *Polistes helveticus* sp. n.) is terminal flagellomere length: malar space (Table 5). Confusion with *Polistes gallicus* or *Polistes hellenicus* is unlikely due to the very different color patterns in both sexes. Males are virtually impossible to confuse with *Polistes biglumis*; however, the very similarly colored females of *Polistes helveticus* sp. n. and *Polistes biglumis* are likely to be confused in specimens of *Polistes helveticus* sp. n. with an exceptionally developed epicnemial carina. For such cases, we provide the ratio malar space: lateral ocelli distance, which fully separates the two.

Since most collected specimens labeled as “*Polistes bischoffi*” are presumed to belong to *Polistes helveticus* sp. n., rather than to *Polistes bischoffi* Weyrauch, 1937 (revised status), at least in Central European museums, their identity must be checked. In fact, according to the CSCF (www.cscf.ch; in litt.) there are about 450 individuals of *Polistes helveticus* sp. n. from Switzerland deposited in Swiss museums, but only very few (< 10) individuals of *Polistes bischoffi*, at least before the material of the present study was deposited. A similar situation may apply to other Central European museums, especially in Austria and Germany. In contrast, the relatively few individuals labeled as “*Polistes bischoffi*” that we examined from Southern Europe (Greece, Italy, Southern France) are, in fact, determined correctly (mostly by Josef Gusenleitner).

The specimen that we have chosen as the holotype of *Polistes helveticus* sp. n. clearly belongs to *Polistes helveticus* sp. n. according to molecular and morphological analyses. According to its body measurements, however, it lies in an area of overlap with *Polistes gallicus* (Fig. 3a, c [H]). Unfortunately, it is the only specimen that was both intact and suitable for molecular analyses.

##### Distribution.

Fig. 11 only shows the distribution of the specimens examined within this study. Other possible records of *Polistes helveticus* (still under the name “*bischoffi*”) not shown in Fig. 11 include localities in Austria (Gusenleitner 1995: 165; 1998: 497), Belgium (Baugnée 1996), Czech Republic and Slovakia (Dvořák et al. 2006), France (Graf 1961), Germany (Mauss 2001, Schmid-Egger and Treiber 1989), and Luxembourg (Schneider et al. 1998).

Nevertheless, Fig. 11 indicates a geographical separation between *Polistes helveticus* sp. n. (in the north) and *Polistes bischoffi* (in the south), leaving only a small area of overlap. Real syntopy (habitat sharing) between the two species has thus far only been assessed in Switzerland but both species also occur sympatrically in Austria. Furthermore, the verified range of *Polistes helveticus* sp. n. (Fig. 11) is distinctly smaller than that of *Polistes bischoffi*, although *Polistes helveticus* sp. n. is considered to be in a period of expansion (Dvořák et al. 2006, Mauss 2001).

##### Ecology.

In Switzerland, *Polistes helveticus* sp. n. is widespread (Fig. 11), usually occurring in wet habitats such as floodplains, fens, bogs, and pits (gravel, sand). The altitudinal records range from 200 m above sea level (Le Champ-près-Froges, France) for a female (individual RN0378) to 980 m a.s.l. (Muggio, Canton of Ticino, CH) for a male (RN0387). The seasonal records range from 02 April (Saint-Blaise, CH) for a female to 24 November (Gnadental, Germany) for a female (RN0283), but most individuals of both sexes are recorded in July and August (CSCF, in litt.). The earliest record for a male is on 21 July (Jestetten, Germany), the latest (RN0157) on 01 October (Courroux, CH). Nests are usually attached below 40 cm to vertical stems (Ø 2–3 cm) of reed, alder, willow and other plants (Kofler 2005; Neumeyer et al. 2011).

##### Etymology.

The Latin adjective “helveticus -a -um” means Swiss. The name *Polistes helveticus* sp. n. pays tribute to the country where the species was first discovered, when a female (Theodor Steck leg., NHMB coll.) was found in Bätterkinden (canton of Berne) in August 1882.

##### Material examined.

Holotype ♀ (RN0139): SWITZERLAND, CANTON ZURICH, **Schwerzenbach, Böschen**, 47°22'21.67"N, 08°40'01.43"E, 436 m, 11 Aug 2010, fen rotation fallow, Rainer Neumeyer leg., NMBE coll.

Original Paralectotype (Weyrauch 1939: 164) of *Polistes bischoffi* Weyrauch, 1937: 1 ♂ (RN0325): Switzerland, CANTON ZURICH, **Glattbrugg**, 8 Sep 1937, Wolfgang Weyrauch leg., HUMCZ coll., labeled as follows: 1. “W. Weyrauch” [printed], “Glattbrugg bei Zürich, 8.9.37” [handwritten]. 2. “Polistula bischoffi Weyrauch 1938” [handwritten]; 3. “Glattbrug [sic] nr Zurich Switzerland [handwritten; most probably added posteriorly]; 4. “Polistes bischoffi ♂ Weyr. paratype.” [handwritten; probably added posteriorly].

Paratypes: 1 ♀ (RN0412): Austria, Burgenland, **Stadtschlaining**, **Goberling**, 18 May 1990, Michael Madl leg., NHMW coll.; 1 ♀ (RN0399): Czech Republic, South Bohemia, **Stachy, Úbislav**, 12 Oct 2005, village, Libor Dvořák leg., LD coll.; 1 ♂ (RN0398): **Vlkov nad Lužnicí**, 18 Sep 2004, P. Bogusch leg., LD coll.; 1 ♀ (RN0378): France, Isère, **Le Champ-près-Froges**, 200 m, 15 Aug 2003, sand pit, J. & I. Smit leg., JS coll.; 1 ♀ (RN0282) + 1 ♂ (RN0284): Germany, Baden-Württemberg, **Blaustein**, **Lautertal westl. Weidach**, 30 Aug 1988, Volker Mauss leg., RN coll.; 1 ♀ (RN0362): **Kaiserstuhl**, 30 Jun 1938, J.P. Wolf leg., ETHZ coll.; 1 ♀ (RN0283): **Michelfeld, Gnadental**, 24 Nov 1992, Volker Mauss leg., RN coll.; 1 ♀ (RN0388): Bavaria, **Weichs, Weichser Moos**, 48°22'55.2"N, 11°25'58.8"E, fen, 31 Jul 1991, Stephan Blank leg., SDEI coll.; 1 ♂ (RN0162): Principality of Liechtenstein, **Ruggell**, 47°13'56.98"N, 09°39'43.61"E, 444 m, 27 Aug 1996, dam, Simon Bieri leg., ETHZ coll.; 1 ♂ (RN0164): **Schaan**, 47°11'02.53"N, 09°31'29.11"E, 455 m, 24 Sep 1997, Simon Bieri leg., ETHZ coll.; 1 ♀ (RN0400): Slovakia, Trenčín Region, **Bzince pod Javorinou**, 06 Aug 2004, glade, Libor Dvořák leg., LD coll.; 1 ♀ (RN0230): SWITZERLAND, CANTON BASLE-City, **Basel, Badischer Bahnhof**, 47°34'50.12"N, 07°36'07.63"E, 255 m, 18 Aug 1995, ruderal field, Rainer Neumeyer leg., RN coll.; 1 ♀ (RN0377): CANTON BERNE, **Bern**, 22 Jul 1883, Theodor Steck leg., NMBE coll.; 1 ♀ (RN0374) + 2 ♂ (RN0375, RN0376): **Bätterkinden**, 24 Aug 1887, Theodor Steck leg., NMBE coll.; 1 ♂ (RN0161): **Gampelen, Seewald**, 16 Aug 1994, Richard Vernier leg., MHNN coll.; 1 ♂ (RN0158): CANTON Jura, **Damphreux, Les Coeudres**, 47°28'23.54"N, 07°06'34.74"E, 430 m, 22 Aug 2003, Christian Monnerat leg., MHNN coll.; 1 ♂ (RN0157): **Courroux, Le Quenet**, 47°22'46.78"N, 07°21'40.20"E, 510 m, 01 Oct 2004, Christian Monnerat leg., MHNN coll.; 2 ♂ (RN0155, RN0159): **Courtemaîche, La Colombière**, 47°27'48.87"N, 07°02'59.38"E, 390 m, 22 Aug 2003, Christian Monnerat leg., MHNN coll.; 1 ♂ (RN0386): CANTON Ticino, **Meride, Fontana**, 45°53'44"N, 08°56'46"E, 595 m, 24 Aug 1997, Ladislaus Rezbanyai-Reser leg., NML coll.; 1 ♂ (RN0387): **Muggio, Muggiasca**, 45°54'39"N, 09°01'21"E, 980 m, 16 Aug 1996, Ladislaus Rezbanyai-Reser leg., NML coll.; 1 ♀ (RN0394): CANTON Valais, **Brig**, 09 Aug 1916, anon. leg., ETHZ coll.; 2 ♂ (RN0198, RN0199): CANTON Vaud, **Bussigny-près-Lausanne**, 16 Aug 1962, Robert Matthey leg., MZL coll.; 1 ♀ (RN0360) + 1 ♂ (RN0361): **Lausanne**, Aug 1942, Jacques de Beaumont leg., MFNB coll.; 3 ♂ (RN0200, RN0203, RN0204): **Lausanne, Vidy**, 02 Aug 1943, Jacques de Beaumont leg., MZL coll.; 1 ♀ (RN0046): **Noville, Longes Rayes**, 46°23'21.32"N, 06°53'31.03"E, 273 m, 23 Aug 2011, shrubberies, Rainer Neumeyer leg., RN coll.; 5 ♀ (RN0047, RN0048, RN0049, RN0050, RN0051): 46°23'14.28"N, 06°53'34.66"E, 273 m, 23 Aug 2011, tall herbaceous vegetation, Rainer Neumeyer leg., RN coll.; 1 ♂ (RN0160): **Saint-Livres, Les Mossières**, 46°32'02.47"N, 06°21'55.82"E, 700 m, 21 Aug 2002, Christian Monnerat leg., MHNN coll.; 2 ♂ (RN0201, RN0202): **Saint-Sulpice**, Jul 1943, Jacques de Beaumont leg., MZL coll.; 1 ♂ (RN0114): **Villars-sous-Yens, Arborex**, 46°30'11.03"N, 06°25'12.11"E, 510 m, 25 Aug 2010, fen, Christian Monnerat leg., MHNN coll.; 2 ♀ (RN0277, RN0278): CANTON ZUG, **Hünenberg, Rüssspitz**, 47°14'09.40"N, 08°24'39.49"E, 389 m, 10 Jul 2012, fen, Rainer Neumeyer leg., ETHZ coll.; 1 ♀ (RN0279): 47°14'17.60"N, 08°24'27.75"E, 389 m, 20 Aug 2012, fen, Rainer Neumeyer leg., AMNH coll.; 1 ♀ (RN0275): CANTON ZURICH, **Bauma, Fischbach**, 47°23'00.66"N, 08°50'48.30"E, 660 m, 04 Jul 2012, abandoned pit, Rainer Neumeyer leg., RN coll.; 2 ♀ (RN0167, RN0168): 08 Aug 2012, abandoned pit, Rainer Neumeyer leg., CSE coll.; 1 ♂ (RN0152): **Mönchaltorf, Seewisen**, 47°19'17.63"N, 08°41'55.58"E, 436 m, 21 Aug 2010, fen rotation fallow, Rainer Neumeyer leg., AMNH coll.; 1 ♀ (RN0003): **Pfäffikon, Auslikon**, 47°20'42.05"N, 08°47'52.78"E, 539 m, 20 Jun 2011, fen, Rainer Neumeyer leg., RN coll.; 3 ♀ (RN0018, RN0019, RN0020): 47°20'46.94"N, 08°47'50.38"E, 539 m, 10 Aug 2011, fen, Rainer Neumeyer leg., RN coll.; 1 ♀ (RN0078): **Pfäffikon, Irgenhuserriet**, 47°20'59.15"N, 08°47'49.98"E, 539 m, 06 Sep 2011, fen, Rainer Neumeyer leg., RN coll.; 2 ♀ (RN0033, RN0034): **Seegräben, Schlachtmad**, 47°20'23.35"N, 08°46'36.56"E, 537 m, 19 Aug 2011, fen, Rainer Neumeyer leg., RN coll.; 1 ♀ + 2 ♂ (RN0138, RN0153, RN0154): **Schwerzenbach, Böschen**, 47°22'21.67"N, 08°40'01.43"E, 436 m, same nest as holotype, 11 Aug 2010, fen rotation fallow, Rainer Neumeyer leg., NMBE coll.; 1 ♀ (RN0276): **Weiach, Rüteren**, 47°34'03.21"N, 08°26'44.70"E, 365 m, 02 Apr 2005, gravel pit, Rainer Neumeyer leg., RN coll.; 1 ♀ (RN0035): **Wetzikon, Agerstenriet**, 47°20'06.75"N, 08°46'57.43"E, 538 m, 19 Aug 2011, fen, Rainer Neumeyer leg., RN coll.; 2 ♀ (RN0012, RN0013): **Wetzikon, Seeriet**, 47°20'30.24"N, 08°47'10.89"E, 538 m, 05 Aug 2011, fen, Rainer Neumeyer leg., RN coll.; 1 ♀ (RN0081): 09 Sep 2011, fen, Rainer Neumeyer leg., RN coll.; 1 ♀ (RN0014): **Wetzikon, Robenhuserriet**, 47°20'19.16"N, 08°47'02.75"E, 538 m, 05 Aug 2011, fen, Rainer Neumeyer leg., RN coll.; 1 ♀ (RN0017): 47°20'16.20"N, 08°47'34.35"E, 539 m, 05 Aug 2011, fen, Rainer Neumeyer leg., RN coll.; 1 ♂ (RN0163): **Zürich**, < 1900, anon. leg., ETHZ coll.

### c) Key to species of the *Polistes gallicus*-group

The following dichotomous key only applies to the described species of the *gallicus*-group. The text denotes diagnostic traits. However, traits described after a hyphen (–) are those that apply in most cases to species in that half of the couplet, but that may also apply to some species in the alternative half of the same couplet. To determine all European species of *Polistes* including the *dominula*-group, we recommend the keys of Mauss and Treiber (2004), Dvořák and Roberts (2006), and Witt (2009: 129–132), whereby Mauss and Treiber (2004) apply to Germany only.

**Table:** 

♀	Antenna with 10 flagellomeres. Metasoma with 6 terga	1
♂	Antenna with 11 flagellomeres. Metasoma with 7 terga	6

Females:

**Table:** 

1	Malar space usually black, mandible spotted yellow (Fig. 12g, h). If yellow area present on malar space, then always smaller than area on mandible	2
–	Malar space yellow, mandible usually black. If yellow area on mandible, then always smaller than area on malar space	*dominula*-group
2	Flagellum black on upper side, bright yellow to orange on lower side (Fig. 12c). – Metacoxa black. Mesoscutum usually without yellow spot (Fig. 12k). Clypeus with central, black spot (Fig. 12h) or more often with black, horizontal bar (Fig. 12g). Hypopygium entirely black	3
–	Flagellum bright yellow to orange (Fig. 12d) or faintly darkened on upper side, but never black	4
3	Epicnemial carina distinct, marking an abrupt change in sculpture between coarse mesepisternum and smooth epicnemium (Fig. 12a). Malar space: lateral ocelli distance 1.22–1.76. – Mesoscutum with relatively long pubescence (Fig. 12a)	*Polistes biglumis*
–	Epicnemial carina reduced (Fig. 12b) or absent. Change in sculpture between mesepisternum and epicnemium often gradual (Fig. 12b). Malar space: lateral ocelli distance 0.87–1.19	*Polistes helveticus* sp. n.
4	Epicnemial carina reduced (Fig. 12b) or absent. Change in sculpture between mesepisternum and epicnemium frequently gradual (Fig. 12b). Terminal flagellomere length: malar space 1.00–1.24. – Clypeus yellow, although almost never entirely so, frequently with central black spot (Fig. 12h), occasionally even with horizontal black band (Fig. 12g). Mesoscutum usually without yellow spot (Fig. 12k), only occasionally with pair of yellow spots (Fig. 12i). Metacoxa frequently black, only occasionally spotted yellow. Hypopygium entirely black	*Polistes bischoffi*
–	Epicnemial carina distinct (Fig. 12a) or reduced, usually marking a sudden change in sculpture between coarse mesepisternum and smooth epicnemium (Fig. 12a). Terminal flagellomere length: malar space 0.74–1.11. – Clypeus yellow, with or without central, black spot (Fig. 12h), but almost never with horizontal black band. Metacoxa frequently spotted yellow, only occasionally black	5
5	Hypopygium entirely black, only rarely spotted yellow at tip. Lateral part of propodeum with yellow spot usually more than half the size of mesopleural spot (Fig. 6c). Mesoscutum frequently with pair of yellow spots (Fig. 12i). Pronotum with paired longitudinal yellow stripes along posterior margin usually not reaching yellow cross stripe on pronotal collar (Fig. 12i)	*Polistes gallicus*
–	Hypopygium frequently orange and yellow at tip, only occasionally entirely black. Lateral part of propodeum with yellow spot of less than half the size of mesopleural spot, if present at all. Mesoscutum only occasionally with pair of yellow spots. Pronotum with longitudinal yellow stripes often reaching yellow cross stripe on pronotal collar (Fig. 12k)	*Polistes hellenicus*

Males:

**Table:** 

6	Gena convex in dorsal view (Fig. 12l)	7
–	Gena converging in dorsal view (Fig. 12m)	8
7	Flagellum black on dorsal side, bright yellow to orange on ventral side (Fig. 12e). Frontal groove and lateral clypeal ridges reduced or absent. Terminal flagellomere length: terminal flagellomere breadth 1.64–2.68. – Black area of upper frons often with bat-shaped yellow spot	*Polistes biglumis*
–	Flagellum bright yellow to orange all around (Fig. 12f) in most species. If not, frontal groove and lateral clypeal ridges distinct, and terminal flagellomere length: terminal flagellomere breadth > 2.5	*dominula*-group (in part)
8	Epicnemium and mesosternum black (Fig. 8), seldom with pair of elongate yellow spots between pro- and mesocoxa	*Polistes hellenicus*
–	Epicnemium and mesosternum entirely yellow (Fig. 7d)	9
9	Flagellum black or blackish dorsally, bright yellow to orange ventrally (Fig. 12e)	10
–	Flagellum entirely bright yellow to orange (Fig. 12f) or faintly darkened dorsally. – Frontal groove reduced	11
10	Frontal groove and lateral clypeal ridges reduced. Terminal flagellomere length: terminal flagellomere breadth 1.83–2.78	*Polistes helveticus* sp. n.
–	Frontal groove and lateral clypeal ridges very distinct. Terminal flagellomere length: terminal flagellomere breadth > 2.5	*dominula*-group (*associus*)
11	Clypeus with moderate but distinct lateral ridges. Mesoscutum often with pair of drop-shaped yellow spots (Fig. 12i). Metacoxa frequently spotted yellow dorsally. Terminal flagellomere length: malar space 1.28–2.21	*Polistes gallicus*
–	Clypeus with reduced or absent lateral ridges. Mesoscutum seldom with pair of drop-shaped yellow spots. Metacoxa seldom spotted yellow dorsally. Terminal flagellomere length: malar space 1.93–2.75	*Polistes bischoffi*

Although it probably belongs to the *gallicus*-group too, the ambiguous taxon from Greece (and possibly elsewhere) referred to as “*Polistes* sp. aff. *gallicus*” is not included in this key because there was not enough material available to examine.

## Discussion

**Status of *Polistes bischoffi* and *Polistes helveticus* sp. n.** Our study unambiguously demonstrates that two distinct species are included within what has been so far considered as *Polistes bischoffi* Weyrauch, 1937: a light colored species (*Polistes bischoffi*) with a Southern European to West Asian distribution, and a dark, Central European species described here as *Polistes helveticus* sp. n.

The distinctivness of these taxa (*Polistes bischoffi*, *Polistes helveticus* sp. n.) is revealed in analyses of two independent molecular markers (COX1, ITS1), as well as in our morphometric analyses. Moreover, *Polistes helveticus* sp. n. is probably closely related to *Polistes bischoffi* (as suggested by the reduced epicnemial carina and the association with wetlands) and occurs in the same habitats, sometimes syntopically, but appears not to interbreed (Neumeyer et al. 2011). Taken together, these results suggest that three independent criteria are met to reveal the presence of a new species: molecules, morphology, and syntopy without interbreeding (Neumeyer et al. 2011).

The unnoticed presence of a cryptic species in Europe is surprising and calls for an explanation. Interestingly, the first record for *Polistes bischoffi* Weyrauch, 1937 (rev. status) in Switzerland refers to two individuals (RN0170, RN0171) found in 1927 in Versoix near Geneva, in the extreme southwest of Switzerland where *Polistes gallicus* is known to have occurred before 1900 (cf. our examined individual RN0208). The second Swiss record (RN0156) of *Polistes bischoffi* is from Chabrey on Lake Neuchâtel in 1992, and the third (RN0169) from Regensdorf near Zurich in 1997, all together suggesting a recent range expansion from the southwestern to the northeastern part of the Swiss midlands, where *Polistes gallicus* still does not occur. We hypothesize that *Polistes bischoffi* was originally present but remained undetected within the range of the superficially similar *Polistes gallicus*, and became conspicuous only after it expanded beyond the range of *Polistes gallicus*, possibly due to global warming.

**Morphometry.** By applying multivariate ratio analysis (MRA) most taxa of the *gallicus*-group are rather well differentiated (Fig. 3), with the exception of *Polistes gallicus* and *Polistes hellenicus*. The use of further measurements may have resulted in better differentiation between these two species, as the addition of characters has indeed improved the separation of sibling species in some other Hymenoptera (e.g., Kenis and Mills 1998, Villemant et al. 2007). However, such analyses are beyond the scope of this study, as *Polistes bischoffi* and *Polistes helveticus* sp. n. were clearly separated by the first shape PC (Fig. 4).

The latter two species were of comparable size, so allometric variation did not interfere with the interpretation of the data. This was also unlikely to bias the differentiation of *Polistes biglumis*, although this large species accounted for the rather strong correlative pattern between size and shape in the shape PCA of all five species of the *gallicus*-group (Figs 3c, d). Since *Polistes biglumis* could clearly be separated from the rest of the *gallicus*-group by qualitative morphological characters and molecular analysis, the morphometric separation was assumed to be based on “true” shape differences and not merely on an indirect size effect. Hence, we see no need to correct the best separating ratio (Tab. 5) for allometric size effects, although such a procedure has sometimes been suggested (Janzon 1986, Seifert 2008, Bartels et al. 2011).

The LDA ratio extractor revealed ratios that separated some of the species with very little or no overlap (Table 5, species comparisons marked with *). It is noteworthy that these ratios were composed of measurements from widely separated body parts; for instance metatibia length (tib3.l) to malar space (msp.l) was the best ratio for separating the females of *Polistes helveticus* sp. n. from *Polistes bischoffi*. This is in contrast to more commonly used ratios that are calculated from measurements of the same or adjacent body parts, such as eye length to breadth or clypeus height to breadth (e.g., Arens 2011). In our study, such standard ratios are clearly less powerful for separating taxa (compare PCA ratio spectra in Fig. 5), an observation that was also made by László et al. (2013) in their application of MRA to parasitic wasps.

**Utility of the molecular markers.** An important question when using molecular markers to separate closely related species, is whether a clear gap (the barcoding gap) exists between “within-species” distances and “between-species” distances. Buck et al. (2012)’s detailed study of the Nearctic *Fuscopolistes* revealed no barcoding gap within this group for COX1. In fact, half of the species included in their study showed a “negative barcoding gap”, i. e. a situation where “the maximum intraspecific divergence was greater than the distance to the nearest neighbour from another species” (Buck et al. 2012: 34). In our case, the evaluation of such a barcoding gap would strongly depend on our interpretation of the two clades found within *Polistes dominula* with the mitochondrial marker. Two hypotheses can be formulated: firstly, two cryptic species may be present in Central Europe; alternatively, two distinct mitochondrial haplotypes may exist within one single species. As *Polistes dominula* was not the focus of our study, we did not perform any morphometric analyses for this taxon. The nuclear marker ITS1 did not recover these two clades. As a nuclear DNA marker, ITS1 has a lower rate of mutation than the mitochondrial marker, as indicated by the overall smaller genetic distances between species for ITS1 than for COX1. It is therefore possible that ITS1 evolves too slowly to recover the recent divergence between the two clades observed within *Polistes dominula*. However, ITS1 appeared highly suitable for recovering differences between other closely related species. Therefore, we favor the hypothesis that two mitochondrial haplotypes may coexist in Central Europe within *Polistes dominula*, as demonstrated for other species (Avtzis et al. 2008, Arthofer et al. 2010). Possibly, the two haplotypes revealed in *Polistes dominula* reflect two distinct Pleistocene refugia that have facilitated sequence divergence in the mitochondrial marker; divergence time was presumably not long enough for preventing the populations from successfully interbreeding when they entered in contact again. Our example stresses the importance of using additional criteria (morphometry, nuclear DNA) in addition to one single mitochondrial marker (e.g., the universal barcode) to examine the status of populations in systematics. Deep within-species divergences in mitochondrial DNA sequences may be more widespread than hitherto assumed, especially when sampling is done over the entire range of a species (Bergsten et al. 2012, Buck et al. 2012).

In conclusion, our study demonstrates the power of the combined use of morphometrics and molecular markers in unraveling cryptic diversity, as proposed under the framework of integrative taxonomy (Schlick-Steiner et al. 2010). It also stresses the importance of using multiple molecular markers to evaluate the status of unclear taxa.

## Supplementary Material

XML Treatment for Polistes biglumis

XML Treatment for Polistes bischoffi

XML Treatment for Polistes gallicus

XML Treatment for Polistes hellenicus

XML Treatment for Polistes helveticus
